# High-fat diet promotes lipotoxicity in the podocytes of uninephrectomized mice: a targeted lipidomics and kidney podocyte-specific analysis

**DOI:** 10.1038/s41420-025-02419-7

**Published:** 2025-04-23

**Authors:** Se-Hyun Oh, You-Jin Kim, Subin Bae, Hee-Yeon Jung, So-Young Park, Jeong-Hoon Lim, Jang-Hee Cho, Chan-Duck Kim, Sun-Hee Park, Tae-Hwan Kwon, Yong-Jin Kim, Kwang-Hyeon Liu, Yong-Lim Kim

**Affiliations:** 1 Department of Internal Medicine, School of Medicine, Kyungpook National University, Daegu, Republic of Korea; 2 Cell and Matrix Research Institute, Kyungpook National University, Daegu, Republic of Korea; 3 BK21 FOUR Community-Based Intelligent Novel Drug Discovery Education Unit and College of Pharmacy and Research Institute of Pharmaceutical Sciences, Kyungpook National University, Daegu, Republic of Korea; 4 Mass Spectrometry Based Convergence Research Institute, Kyungpook National University, Daegu, Republic of Korea; 5 Department of Biochemistry and Cell Biology, School of Medicine, Kyungpook National University, Daegu, Republic of Korea; 6 Department of Pathology, School of Medicine, Kyungpook National University, Kyungpook National University Hospital, Daegu, Republic of Korea

**Keywords:** Lipidomics, End-stage renal disease

## Abstract

Abnormal lipid metabolism is an independent risk factor for kidney injury, significantly altering the associated gene expression, particularly in single kidney models. This study investigates the impact of high-fat diet-induced lipid metabolism on podocyte injury in uninephrectomized mice. Using targeted lipidomics analysis and podocyte-specific assays, the modification of lipid profiles attributed to a high-fat diet and the development of podocyte injury caused by lipid metabolism in mice that underwent unilateral nephrectomy were examined. Mice that underwent unilateral nephrectomy and were fed with a high-fat diet for 13 weeks exhibited progressive renal dysfunction, including the accumulation of lipid droplets in podocytes, vacuolization of tubular cells, and glomerular hypertrophy. Liquid chromatography-triple quadrupole mass spectrometry confirmed a significant increase in cholesteryl ester 20:4 levels in the podocytes of these mice. In vitro, cholesteryl ester 20:4 treatment reduced mitochondrial respiration capacity and mitochondrial glycolysis in podocytes. Furthermore, the treatment led to alterations in the protein expression levels associated with lipid metabolism and transport, mitochondrial activity, and autophagy, including ATP binding cassette subfamily A member 1 (ABCA1), carnitine palmitoyltransferase 1 A (CPT1A), acyl-CoA cholesterol acyltransferase (ACAT), nuclear respiratory factor ½ (NRF½), dynamin-1-like protein (DRP1), and p62. Transcriptome sequencing analysis revealed impaired gene expression, which was associated with the progression of renal fibrosis in unilateral nephrectomy mice with a high-fat diet. Specifically, the expression of matrix metalloproteinases and collagen genes, including fibronectin and collagen IV, was upregulated, indicating fibrosis progression. In conclusion, lipidomics analysis identifies cholesteryl ester 20:4 as a key lipid metabolite accumulating in podocytes, which is associated with mitochondrial dysfunction and abnormal autophagy. This accumulation potentially contributes to structural and functional deterioration in the kidney and highlights its role in kidney damage and its potential as a therapeutic target in metabolic kidney diseases.

## Introduction

Living kidney donors can be at high risk of chronic kidney disease (CKD) or end-stage kidney disease (ESKD) [1]. Donors with obesity have a significantly higher incidence of ESKD than normal-weight donors [2, 3]. Obesity is a major risk factor for CKD, independent of its association with hypertension, diabetes, and dyslipidemia [4]. The accumulation of lipid vacuoles in podocytes, mesangial cells, and proximal tubular epithelial cells is another significant characteristic of lipid-related kidney disease. This phenomenon indicates abnormal lipid metabolism and lipotoxicity, which can be a significant cause of renal dysfunction [5].

Lipidomics analyses have demonstrated an association between increased levels of specific lipid metabolites, such as ceramide and sphingosine-1-phosphate, in the blood and kidney damage induced by inflammation and oxidative stress in the kidneys [6]. Abnormal lipid metabolism exacerbated renal fibrosis via the xanthine oxide pathway, one of the cholesterol accumulation-mediated fibrotic pathways, in mice with unilateral kidney who were fed with a high-cholesterol diet. The inhibition of this pathway induced ATP binding cassette subfamily A member 1 (ABCA1), sterol regulatory element binding transcription factor 2 (SREBP2), and 3-hydroxy-3-methylglutaryl-CoA reductase (HMGCR) regulation and reduced fibrosis [7]. However, the association between specific lipid metabolite accumulation and the development of CKD in unilateral kidneys remains unclear.

The present study aimed to identify the mechanism of kidney damage by targeting specific lipid metabolites via lipidomics analysis of podocytes in mice with unilateral kidney who were fed a high-fat diet.

## Results

### In a unilateral kidney model, a high-fat diet impaired kidney function and lipid accumulation in podocytes

**Fig. 1:**
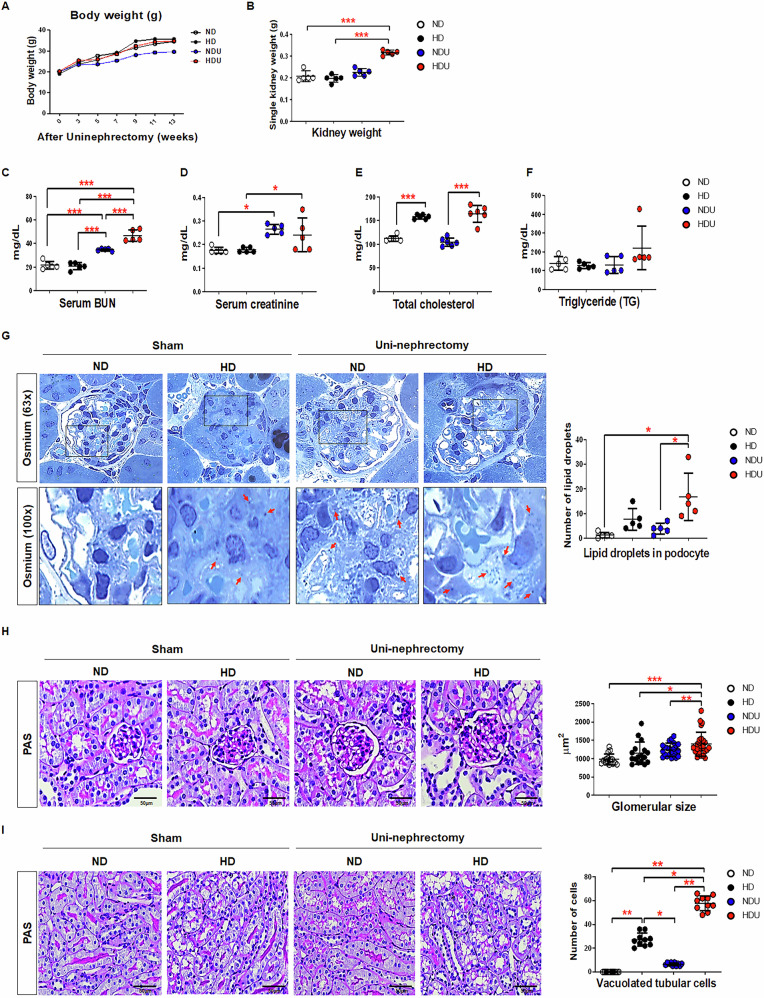
Renal function and lipid accumulation induced by a high-fat diet in a unilateral kidney injury model. Biochemical analysis and renal histology in mice with bi- or unilateral kidneys after 13 weeks of dietary intervention. **A** Body weight measured at the indicated time points. **B** Kidney weight normalized for body weight. Bar graphs showed the BUN (**C**), creatinine (**D**), total cholesterol (**E**), and triglyceride (**F**) levels in the serum were determined. *n* = 5–6 each group. **G** Representative histology of the glomeruli via osmium staining (63×). Lower panes are enlarged images of the boxed areas (100×). The arrows indicate lipid droplets in podocytes. Bar graph summarizes the number of lipid droplets in podocytes. *n* = 5 each group. **H** Representative histological photomicrograph of glomeruli on periodic acid–Schiff (PAS) staining (40×) and bar graph summarizing the glomerular area (µm^2^). *n* = 15 each group. **I** Representative histological photomicrograph of proximal tubules on PAS staining (40×) and bar graph summarizing the number of vacuolated tubular cells. *n* = 10 each group. Each bar represents the mean ± SEM. *p < 0.05, **p < 0.01, ***p < 0.001. ND sham mice fed with a normal diet, HD sham mice fed with a high-fat diet, NDU uninephrectomized mice fed with a normal diet, HDU uninephrectomized mice fed with a high-fat diet.

The body weight of mice did not significantly differ between groups, except in the normal diet and uninephrectomy (NDU) group, which had the lowest weight (Fig. 1A). The kidney weight of the high-fat diet and uninephrectomy (HDU) group significantly increased (Fig. 1B). The serum blood urea nitrogen (BUN) and creatinine levels of the uninephrectomy (UN) groups (NDU and HDU) increased compared with that of the sham group (normal diet [ND] and high-fat diet [HD]) (Fig. 1C, D). The HD and HDU groups had increased total cholesterol levels compared to those of the ND group (Fig. 1E). However, there was no difference in triglyceride levels between the groups (Fig. 1F). These findings suggest that UN and HD induce kidney dysfunction. Furthermore, as observed in the HDU group, HD appears to aggravate UN-induced kidney function.

The lipid droplets of the HD group increased compared to those of the ND group. Moreover, the HDU group had lipid droplets and vacuolation in the podocyte cytoplasm, indicating increased lipid accumulation owing to HD and UN (Fig. 1G). The size of the kidney glomeruli of the HDU group significantly increased compared to that of the ND and HD groups (Fig. 1H). Tubular vacuoles in the HD and HDU groups significantly increased compared to those in the ND group. In particular, the HDU group had increased vacuole formation compared to the HD group (Fig. 1I). These observations suggest that lipid accumulation and structural alterations in the kidney, including glomerular hypertrophy and tubular injury, contribute to the progression of kidney damage in this model. The HD not only increases overall kidney damage but also amplifies the impact of UN, resulting in a more severe pathological outcome than either factor alone.

### Long-term high-fat diet increases cholesteryl ester (CE) levels in the podocytes of uninephrectomized mice

**Fig. 2:**
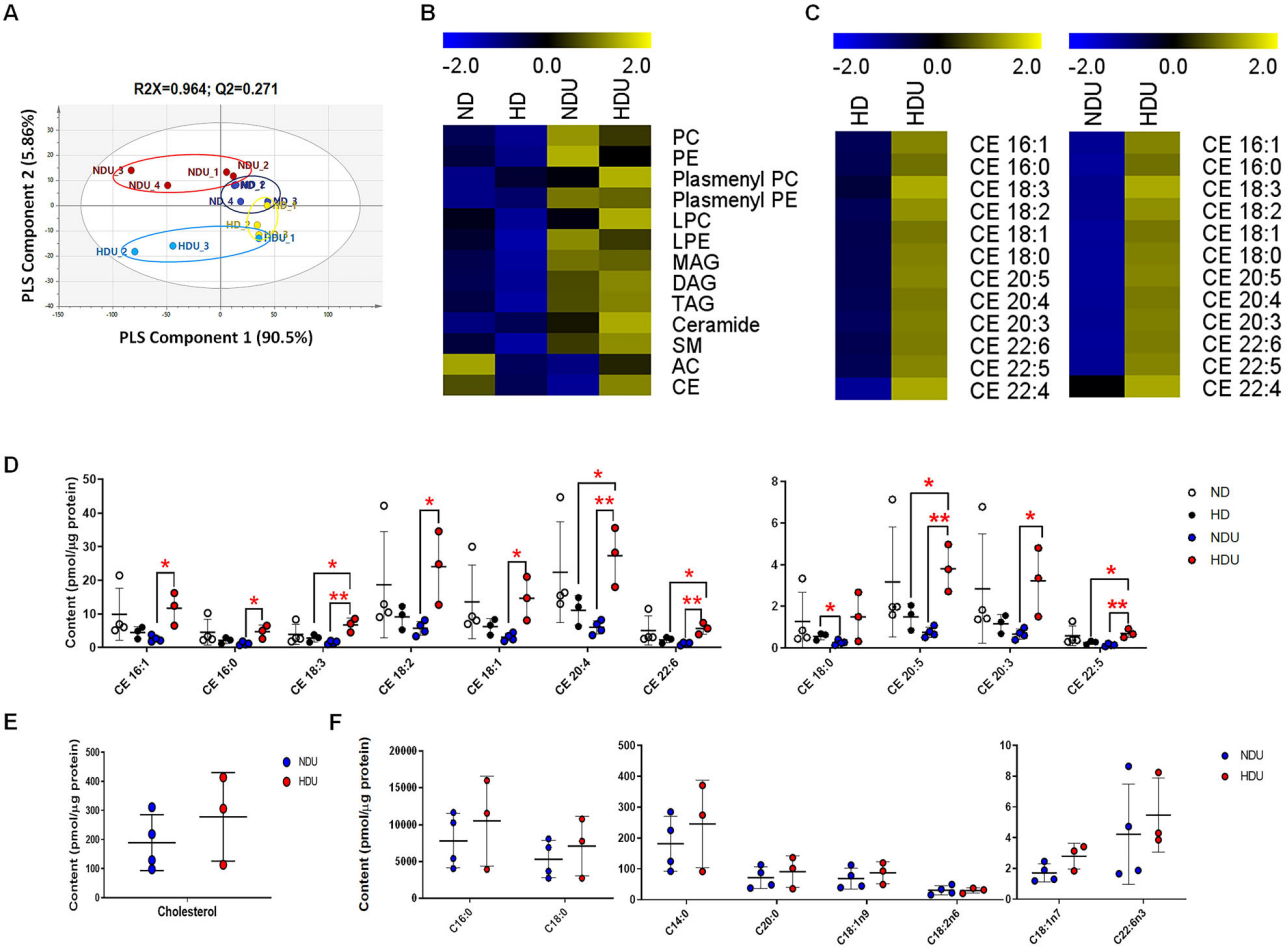
Long-term high-fat diet increases cholesteryl ester levels in the podocytes of uninephrectomized mice. **A** Partial least-squares discriminant analysis score plot obtained via the multivariate analysis of lipid metabolites in mice podocytes. **B** Heatmap of the podocytes lipidome in uninephrectomized mice. **C** Heatmap of cholesteryl ester chains in mice podocytes. Bar graphs showed the content of each cholesteryl ester species (**D**), cholesterol (**E**), and free fatty acid (**F**) species in mice podocytes between groups. Data were expressed as mean ± standard deviation. *p < 0.05, **p < 0.01. *n* = 3–4 each group. ND sham mice fed with a normal diet, HD sham mice fed with a high-fat diet, NDU uninephrectomized mice fed with a normal diet, HDU uninephrectomized mice fed with a high-fat diet.

To identify lipid metabolites specifically accumulating in the unilateral kidney under a high-fat diet, podocytes were isolated from the kidney and subjected to lipidomic analysis. Figure 2A shows the results of multivariate statistical analysis on lipid metabolites in podocytes of the four treatment groups, revealing discernible differences in the score plot among the groups. The total lipid levels quantified in kidney epithelial cells of the ND group did not significantly change in the NDU group (Supplementary Fig. S2A). However, we found that the total levels of phosphatidylcholine (PC), phosphatidylethanolamine (PE), plasmenyl PE, diacylglycerol, and ceramide in podocytes in the NDU group increased compared to that in the ND group (Fig. 2B, Supplementary Fig. S2B). A heatmap analysis showed an increase in 12 types of CE in the HDU group by comparing HD and NDU groups (Fig. 2C). Compared to the NDU group, high-fat diet treatment significantly increased the levels of 10 of 11 CE species (Fig. 2D). Among them, the levels of CE 18:3, CE 20:4, CE 22:5, and CE 22:6 significantly increased in the podocytes of the HDU group (*p* < 0.01). In particular, CE 20:4 levels showed the greatest change. Therefore, the CE 20:4 levels might be used as a toxicity biomarker candidate and could explain kidney damage in uninephrectomized mice fed with HD. These results suggest that HD specifically increases the accumulation of CEs in podocytes, particularly CE 20:4, which may play a critical role in the pathophysiology of kidney damage in uninephrectomized mice.

**Fig. 3:**
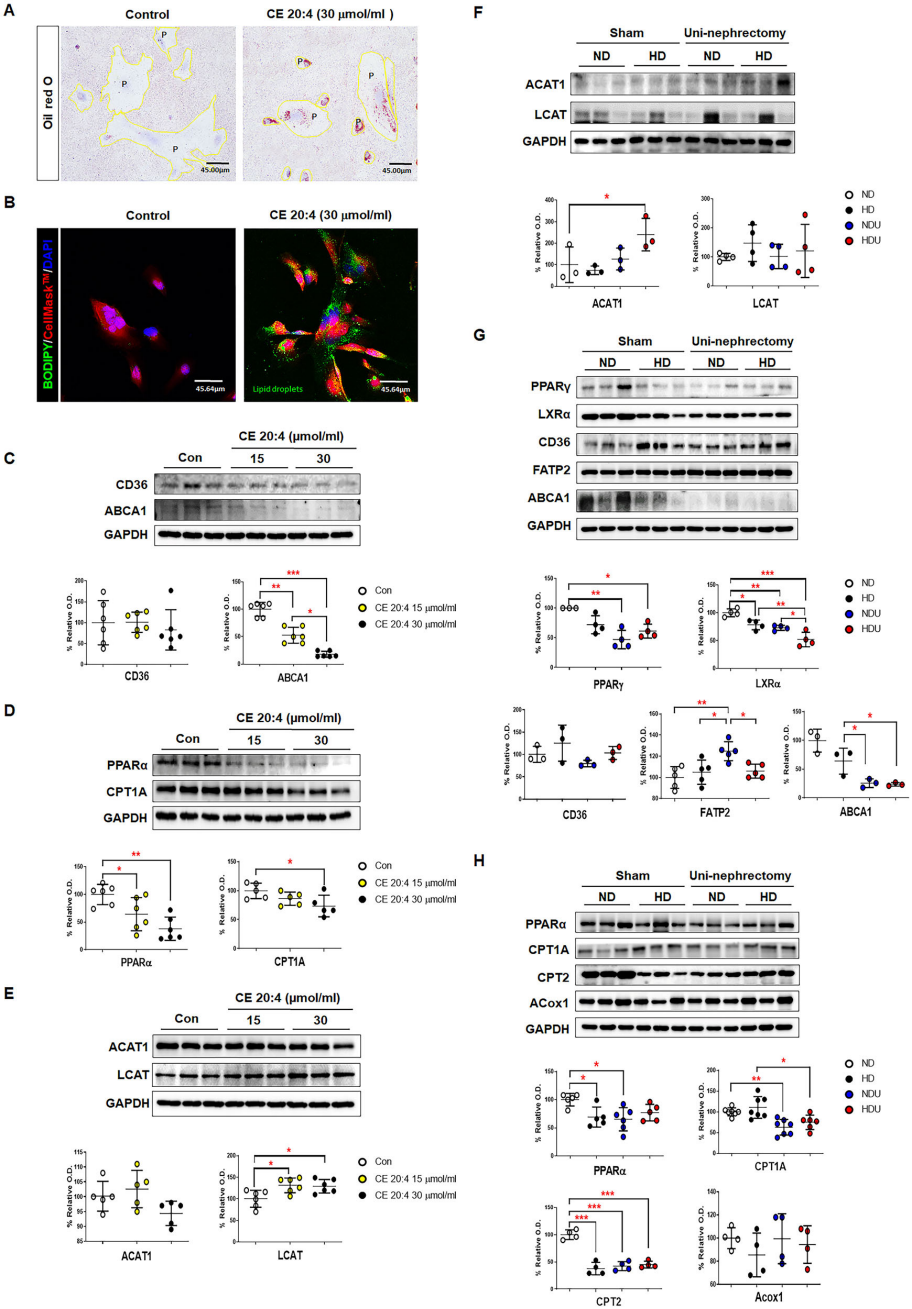
Lipid accumulation and changes in lipid metabolism in CIHP-1 cell lines and the unilateral kidney injury model. **A** Cellular lipid accumulation was confirmed on Oil Red O staining (20×). *n* = 5 each group. **B** Lipid droplets were identified on BODIPY staining. CellMask™ Deep Red shows the plasma membrane in podocytes. (40×). *n* = 5 each group. **C** The expression of lipid transport-associated proteins (CD36 and ABCA1), **D** the expression of β-oxidation-associated proteins (PPARα and CPT1A), and **E** the expression of cholesteryl ester conversion-associated proteins (ACAT and LCAT) in CIHP-1 cells after various treatments were determined via Western blotting analysis. Each bar represents the mean ± SEM. *p < 0.05, **p < 0.01, ***p < 0.001. *n* = 3–6 each group. **F** The expression of cholesteryl ester conversion-associated proteins (ACAT and LCAT), **G** the expression of lipid transport-associated proteins (PPARγ, LXRα, CD36, FATP2, and ABCA1), and **H** the expression of lipid oxidation-associated proteins (PPARα, CPT1A, CPT2, and Acox1) in kidney homogenates and quantitative analysis of protein expression. The band intensities were determined via densitometry using the GAPDH ratios. Each bar represents the mean ± SEM. *p < 0.05, **p < 0.01, ***p < 0.001. *n* = 3–5 each group. ND sham mice fed with a normal diet, HD sham mice fed with a high-fat diet, NDU uninephrectomized mice fed with a normal diet, HDU uninephrectomized mice fed with a high-fat diet.

To determine whether the increase in CE contents in the HDU group resulted from changes in cholesterol and FFA levels, which are CE precursors, these lipids were analyzed. However, cholesterol and FFA levels did not differ significantly between the NDU and HDU groups (Fig. 2E, F). Long-term high-fat diet significantly increased the expression levels of ACAT1 in uninephrectomized mice (Fig. 3F). Based on these findings, the increased levels of CE in uninephrectomized mice fed with a high-fat diet might be attributed to the higher expression of ACAT1. The elevated expression of ACAT1 in the HDU group likely contributes to the increased CE levels in podocytes, indicating that ACAT1 plays a pivotal role in the lipid accumulation observed in HDU mice.

### High-fat diet and CE 20:4 alter the expression of lipid metabolism-related factors in a unilateral kidney model and human podocyte cell line (CIHP-1) cells, respectively

Stimulation with CE 20:4, identified by lipidomics analysis, induced lipid droplet formation in CIHP-1 cells (Fig. 3A, B). CIHP-1 cells treated with CE 20:4 revealed that the expression of the CD36 cluster did not change, and ABCA1 expression significantly reduced with CE 20:4 treatments (Fig. 3C). The expressions of CPT1A and PPARα significantly decreased with CE 20:4 treatments (Fig. 3D). The effects of the CE 20:4-mediated abnormal lipid metabolism on the cholesterol ester conversion enzyme in CIHP-1 cells were confirmed. The expression of ACAT1 did not change, and LCAT significantly increased after CE 20:4 treatment (Fig. 3E). We conclude that CE 20:4 plays a critical role in disrupting lipid metabolism in CIHP-1 cells by inhibiting fatty acid oxidation and promoting cholesterol esterification, which may contribute to lipid accumulation and kidney damage in this model.

The NDU group had a slightly higher ACAT1 expression in the kidney tissue than the ND group, and the HDU group had a significantly higher expression than the other groups. LCAT expression in the HD group was more likely to increase than that in the ND group (Fig. 3F). Notably, the expression of PPAR-γ and LXRα was significantly lower in the NDU and HDU groups than in the ND group. CD36 expression increased slightly in the HDU group, whereas that of FATP2 increased significantly in the NDU group. ABCA1 expression decreased significantly in the NDU and HDU groups compared to the HD group (Fig. 3G). The expression of PPARα, CPT1A, and CPT2 decreased significantly in the NDU and HDU groups compared to the ND group. The expression of ACox1 showed a decreasing trend owing to dietary differences (Fig. 3H). The altered expression of lipid metabolism-related genes in the NDU and HDU groups demonstrates that both a high-fat diet and unilateral nephrectomy affect lipid transport, accumulation, and fatty acid oxidation, contributing to dysregulated lipid metabolism and kidney damage.

### High-fat diet and CE 20:4 respectively induced mitochondrial dysfunction in the unilateral kidney model and CIHP-1 cells

**Fig. 4:**
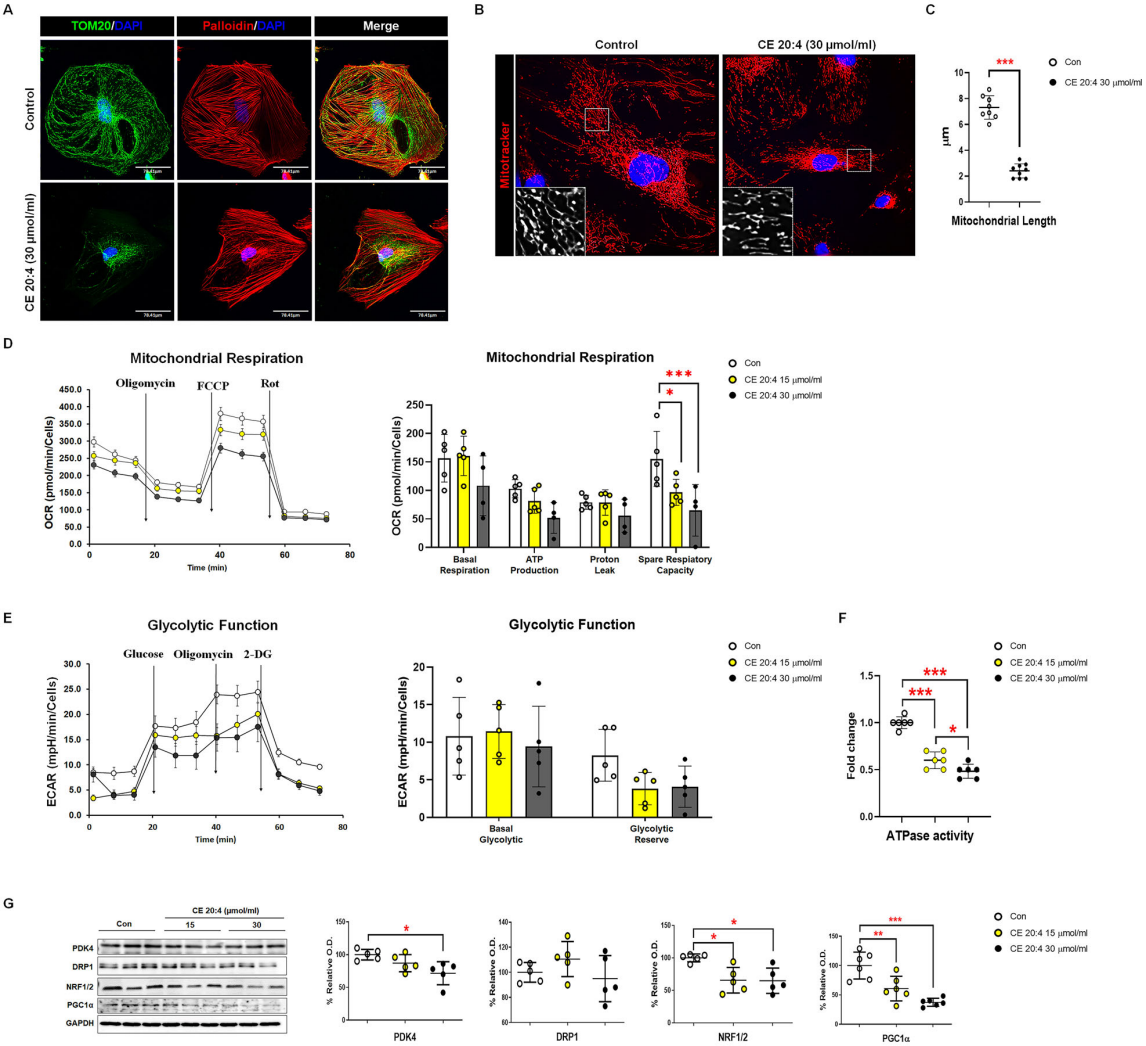
Mitochondrial damage caused by altered lipid metabolism in the CIHP-1 cell line. **A** Degradation of TOM 20 induced by C20:4 treatment. Double-IF staining of TOM20 (green) and phalloidin (red) in CIHP-1 cells (20×). *n* = 5 each group. **B** Mitochondrial images in CIHP-1 cells stained with MitoTracker Red (red) and nucleus (DAPI, blue) (20×). *n* = 5 each group. **C** Quantification of mitochondrial length. **D** Measurement of mitochondrial oxygen consumption ratio and **E** extracellular acidification rates (ECAR) in CIHP-1 cells, *n* = 4-5 each group. **F** Measurement of mitochondrial ATPase activity in CIHP-1 cells, *n* = 6 each group. **G** The expression of mitochondria-associated proteins (PDK4, DRP1, NRF1/2, and PGC1α) in CIHP-1 cells after various treatments was determined via Western blotting analysis. *n* = 5 each group. Each bar represents the mean ± SEM. *p <  0.05, **p <  0.01, ***p < 0.001.

The effects of CE 20:4 on mitochondria in CIHP-1 cells were examined. The expression of TOM20 was aggregated around the nucleus in the CE 20:4-treated cells, and its cytoplasmic expression in the CE 20:4-treated cells decreased compared with that in the control cells (Fig. 4A). CIHP-1 cells stimulated with CE 20:4 had a smaller cell size than the control, and mitochondria were shorter (Fig. 4B). Mitochondrial length was shortened in cells stimulated with CE 20:4 (Fig. 4C). The OCR and ECAR were measured to confirm the changes in mitochondrial activity. Cells stimulated with 30 µmol/mL CE 20:4 showed that mitochondrial respiration capacity and mitochondrial glycolysis decreased compared to the control group (Fig. 4D, E). ATPase activity in the CE 20:4-treated group decreased (Fig. 4F). CE 20:4 treatment of CIHP-1 cells led to a decrease in mitochondrial respiration and the expression of biogenesis-related proteins, NRF½ PGC1α, and PDK4. The expression of DRP1, which is involved in mitochondrial division, did not change (Fig. 4G). The CE 20:4 treatment of CIHP-1 cells induced mitochondrial dysfunction, characterized by altered mitochondrial morphology, decreased mitochondrial respiration, glycolysis, and ATPase activity. This was accompanied by reduced expression of mitochondrial biogenesis- and respiration-related proteins, indicating impaired mitochondrial function.

**Fig. 5:**
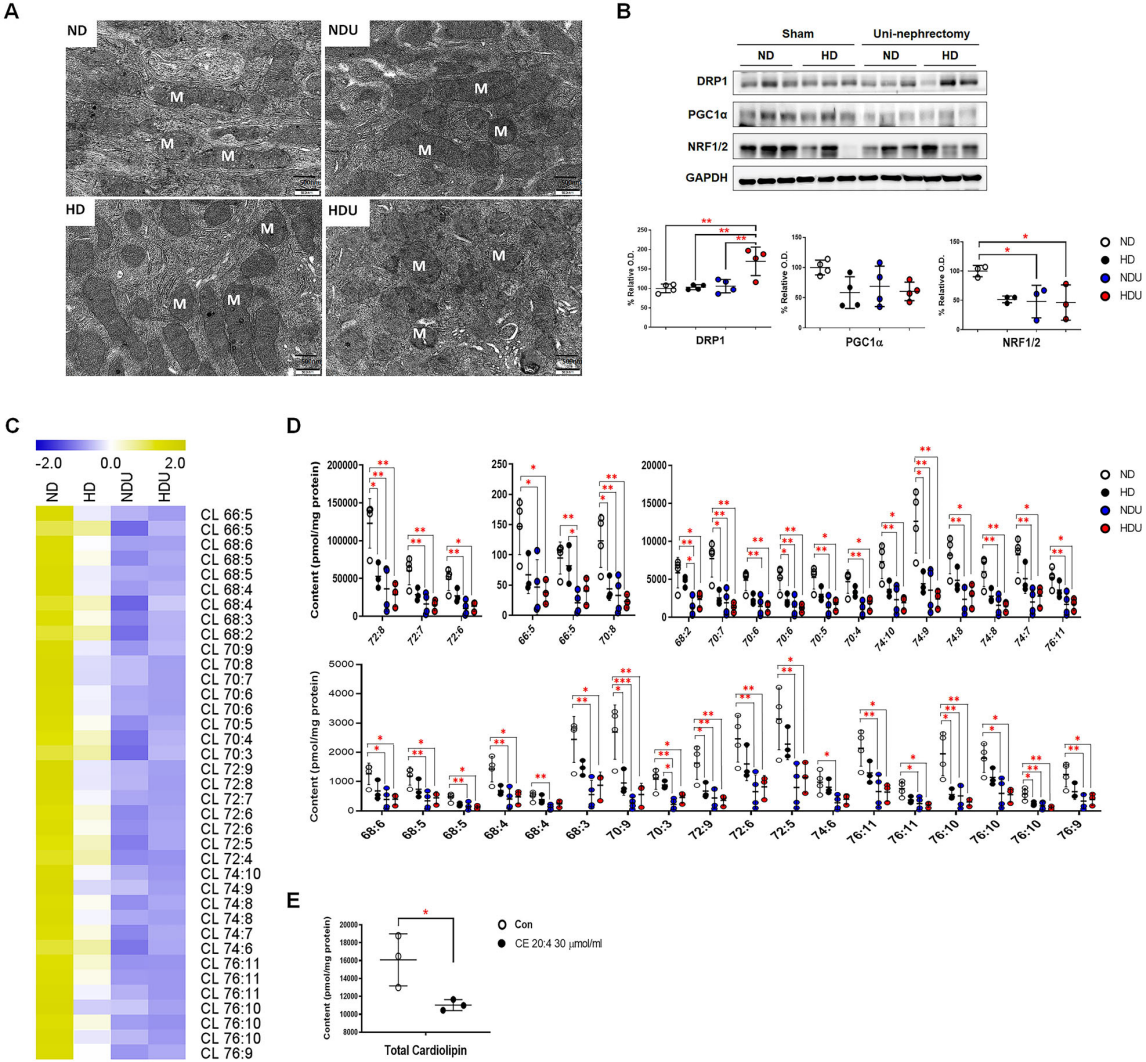
Mitochondrial damage caused by altered lipid metabolism in the unilateral kidney injury model. **A** Transmission electron microscopic (TEM) examination of mitochondrial morphology in the kidney. M, mitochondria. Scale bar = 500 nm. *n* = 5 each group. **B** Western blotting (DRP1, PGC1α, and NRF1/2) in kidney homogenates and quantitative analysis of protein expression. The band intensities were determined via densitometry using the GAPDH ratio. Each bar represents the mean ± SEM. *p < 0.05, **p < 0.01, *n* = 4–5 each group. **C** Heatmap of cardiolipin (CL) in mice kidney tissues. **D** Content of each cardiolipin (CL) species in mice kidney tissues between groups. Data are presented as mean ± standard deviation (SD). *n* = 3 each group. **E** Total CL class content of CHIP-1 cell treated with CE 20:4 (30 µmol/mL). Data were presented as mean ± standard deviation (SD). *n* = 3–4 each group. *p < 0.05, **p < 0.01. ND sham mice fed with a normal diet, HD sham mice fed with a high-fat diet, NDU uninephrectomized mice fed with a normal diet, HDU uninephrectomized mice fed with a high-fat diet.

TEM analysis revealed cristae loss in the mitochondria of kidney tissues in the HD, NDU, and HDU groups (Fig. 5A). DRP1 expression significantly increased in the HDU group, whereas the expression of PGC1α and NRF½ decreased in all groups except the ND group (Fig. 5B). We analyzed the expression of cardiolipin, which is found in the (inner) mitochondria membrane and is essential for the function of enzymes involved in mitochondrial energy metabolism [8]. Heatmap analysis of cardiolipin revealed distinct changes in the four groups (Fig. 5C). Compared with the ND and HD groups, the levels of all cardiolipin species measured decreased in the kidney tissues of uninephrectomized mice (Fig. 5D) (*p* < 0.05). In addition, the total levels of cardiolipin species significantly decreased after CE 20:4 treatment of CIHP-1 cells (Fig. 5E). Reduced levels of cardiolipin and impaired expression of mitochondrial biogenesis-related proteins (PGC1α and NRF½) suggest disrupted mitochondrial integrity and function, linking high-fat diet and CE 20:4 treatment to mitochondrial dysfunction.

### High-fat diet and CE 20:4 respectively trigger autophagy activation in a unilateral kidney model and CIHP-1 cells

**Fig. 6:**
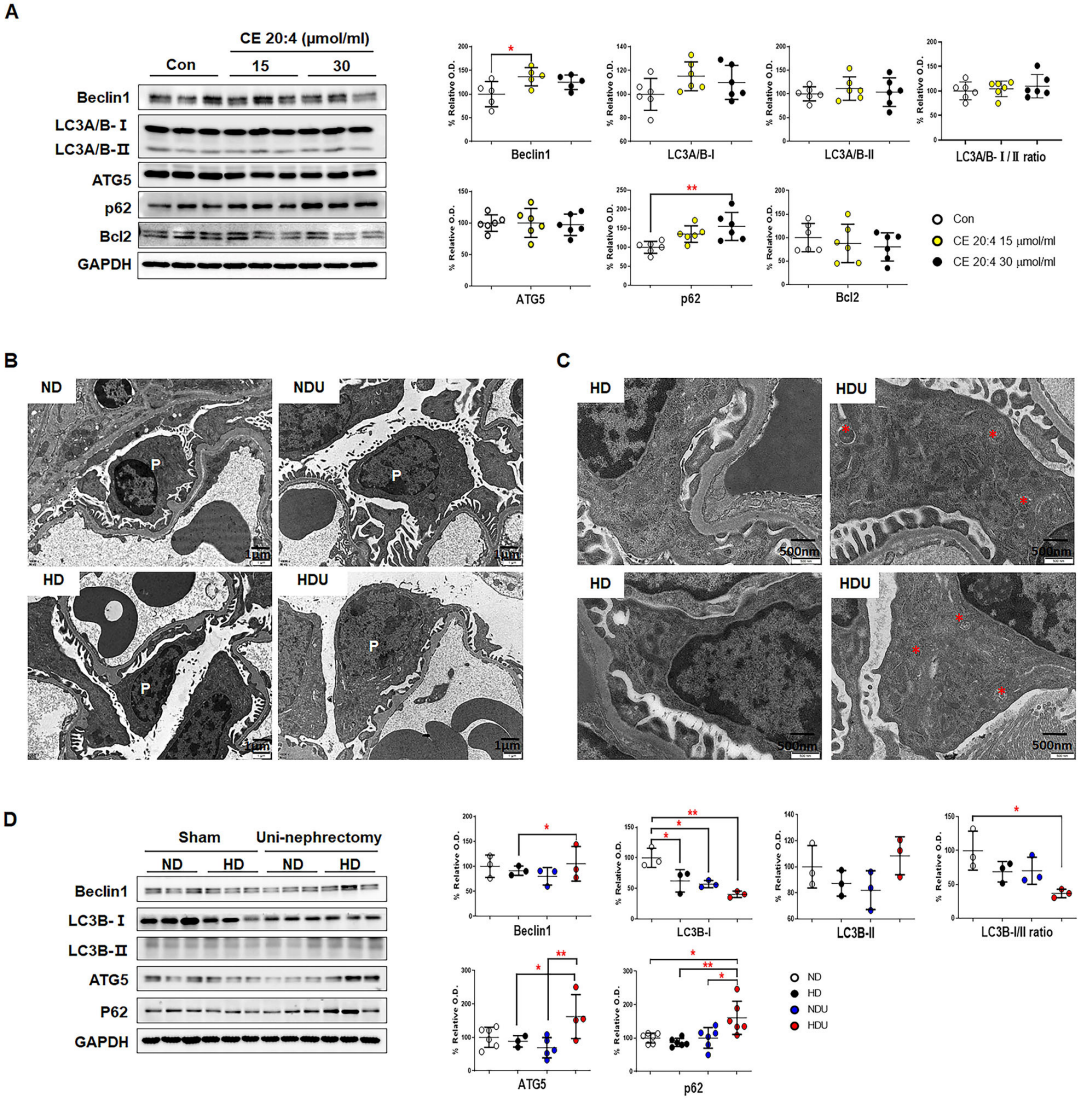
Autophagy activation by lipid accumulation and mitochondrial damage in the CIHP-1 cell line and unilateral renal injury model. **A** The expression of autophagy-associated proteins (Beclin1, LC3-I,II, ATG5, p62, and bcl2) in CIHP-1 cells after various treatments was determined via Western blotting analysis. *p <  0.05, **p <  0.01, *n* = 5 each group. **B** TEM examination of podocyte morphology. Scale bar = 1 µm. *n* = 5 each group. **C** Autophagosomes were detected via TEM in podocytes from the HDU kidney (Asterisk). Scale bar = 500 nm. *n* = 5 each group. **D** The expression of autophagy-associated proteins (Beclin1, LC3B-I, II, ATG5, and p62) in kidney tissues was determined via Western blotting analysis. The band intensities were determined using densitometry with GAPDH ratios. Each bar represents the mean ± SEM. *p < 0.05, **p < 0.01*, n* = 4–6 each group. ND sham mice fed with a *n*ormal diet, HD sham mice fed with a high-fat diet, NDU uninephrectomized mice fed with a normal diet, HDU uninephrectomized mice fed with a high-fat diet.

The expression of Beclin-1, which is involved in the early stages of autophagy, membrane nucleation, and phagophore formation, in the CE 20:4 (15 µmol/mL) treatment group significantly increased compared to that in the control group, whereas the expression of BCL2 decreased. The receptor p62, which moves ubiquitinated cargoes for autophagosome degradation, increased in a concentration-dependent manner in the CE 20:4 treatment group. There was no difference in the expression of ATG5, LC3A/B-I, and LC3B-II in the control group (Fig. 6A).

Based on the assessment of the podocytes of each group with TEM, there was no morphological change in all groups except in the ND group (Fig. 6B). However, autophagosome formation in the HDU group increased compared to that in the HD group (Fig. 6C). The protein expression of Beclin-1, ATG5, and p62, which act on the autophagy pathway, significantly increased in the HDU group. The expression of LC3B-I in all groups except the ND group significantly decreased. However, compared with the sham groups, there was no significant difference in the UN groups (NDU and HDU). The expression of LC3B-II did not change significantly (Fig. 6D). CE 20:4 treatment induced autophagy activation in CIHP-1 cells and the HDU group, as evidenced by increased Beclin-1 and p62 expression.

### High-fat diet causes renal structural changes and fibrosis in the unilateral kidney model

**Fig. 7:**
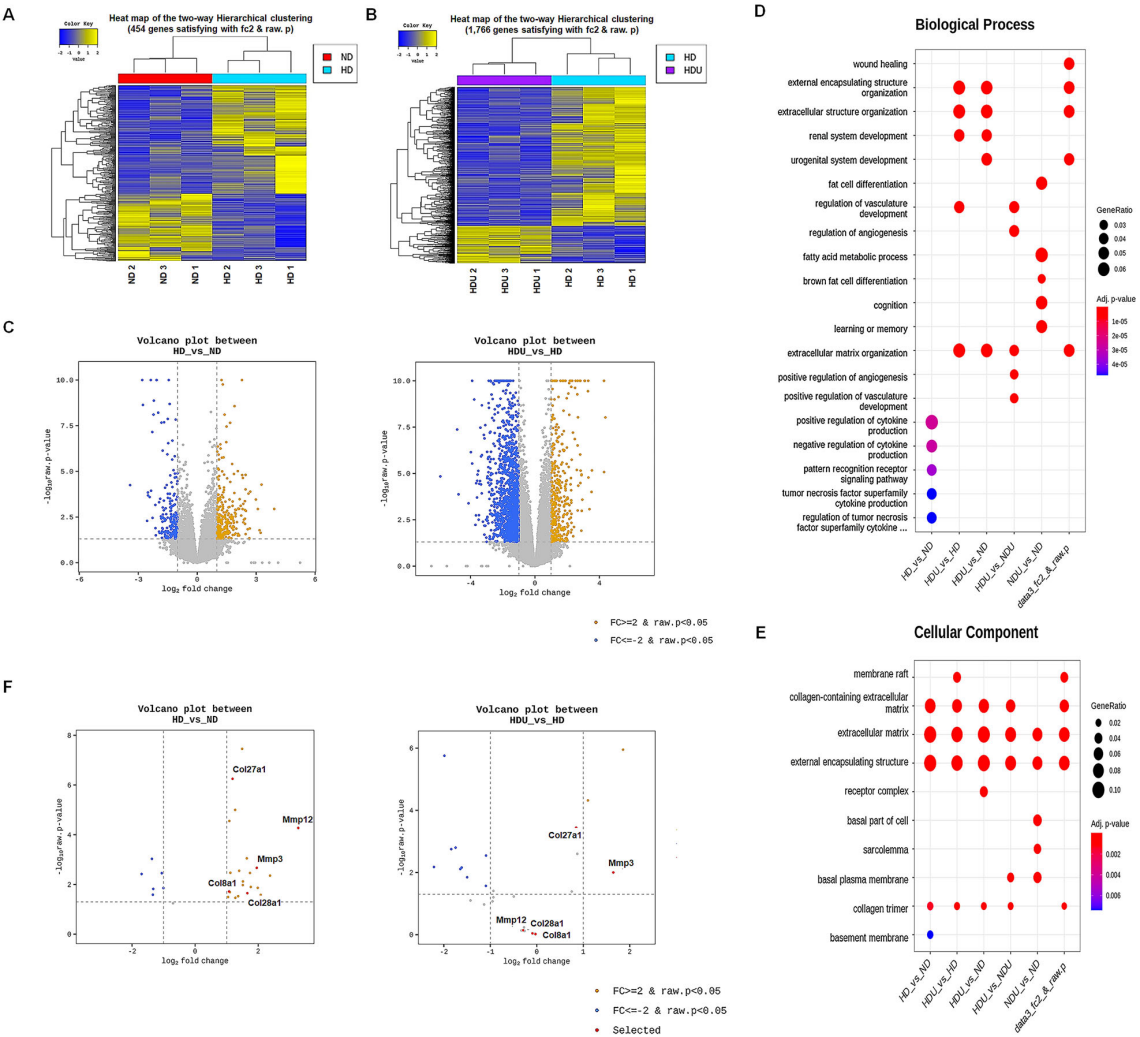
Transcriptome sequence analysis in the unilateral kidney injury model. The heatmap of DEGs enriched in the HD vs. ND groups (**A**) and HDU vs. HD groups (**B**). (*n* = 3 for each group). **C** Volcano plot of DEGs in the HD vs. ND groups and HDU vs. HD groups. The GO enrichment analysis has identified Biological process (**D**) and cellular components (**E**). **F** Volcano plot of DEGs in the ECM, collagen-containing ECM, and wound healing associated-gene through GO analysis in the HD vs. ND groups and HDU vs. HD groups. A fold change (FC) ≥ 2 and *P* value < 0.05 were considered cutoff values for identifying DEGs. *n* = 3 each group. ND sham mice fed with a normal diet, HD sham mice fed with a high-fat diet, HDU uninephrectomized mice fed with a high-fat diet, DEGs differentially expressed genes, GO gene oncology, ECM extracellular matrix.

The association between kidney damage and fibrosis in a high-fat diet-induced unilateral kidney model was investigated using renal pathological and transcriptomic analysis. Differences in the expression were analyzed using a heatmap displaying up- and downregulated differentially expressed genes (DEGs) between the ND and HD groups, as well as between the HD and HDU groups (Fig. 7A, B). A total of 18,829 genes were identified, with 2,577 genes showing significant changes in expression, 454 DEGs in the HD group compared to the ND group, and 1,766 DEGs in the HDU group compared to the HD group. A volcano plot of the DEGs is presented in Fig. 7C.

Using gene ontology (GO) enrichment analysis in terms of biological processes, DEGs were mainly associated with enhanced extracellular matrix (ECM) organization, external encapsulating structure organization, extracellular structure organization, and wound healing (Fig. 7D). For the cellular component functional group, DEGs were enriched in several areas, such as the external side of the ECM, collagen-containing ECM, external encapsulating structure, and the apical part of cells (Fig. 7E).

A volcano plot shows that DEGs associated with ECM organization and wound healing, such as *Mmp12*, *Mmp3*, *Col27a1*, and *Col8a1*, had increased expression in the HD group compared to the ND group. Notably, *Mmp3* and *Col27a1* were further upregulated in the HDU group compared to the HD group (Fig. 7F). These findings indicate that high-fat diet-induced kidney damage is associated with ECM remodeling and fibrosis, which is exacerbated in the unilateral kidney model.

**Fig. 8:**
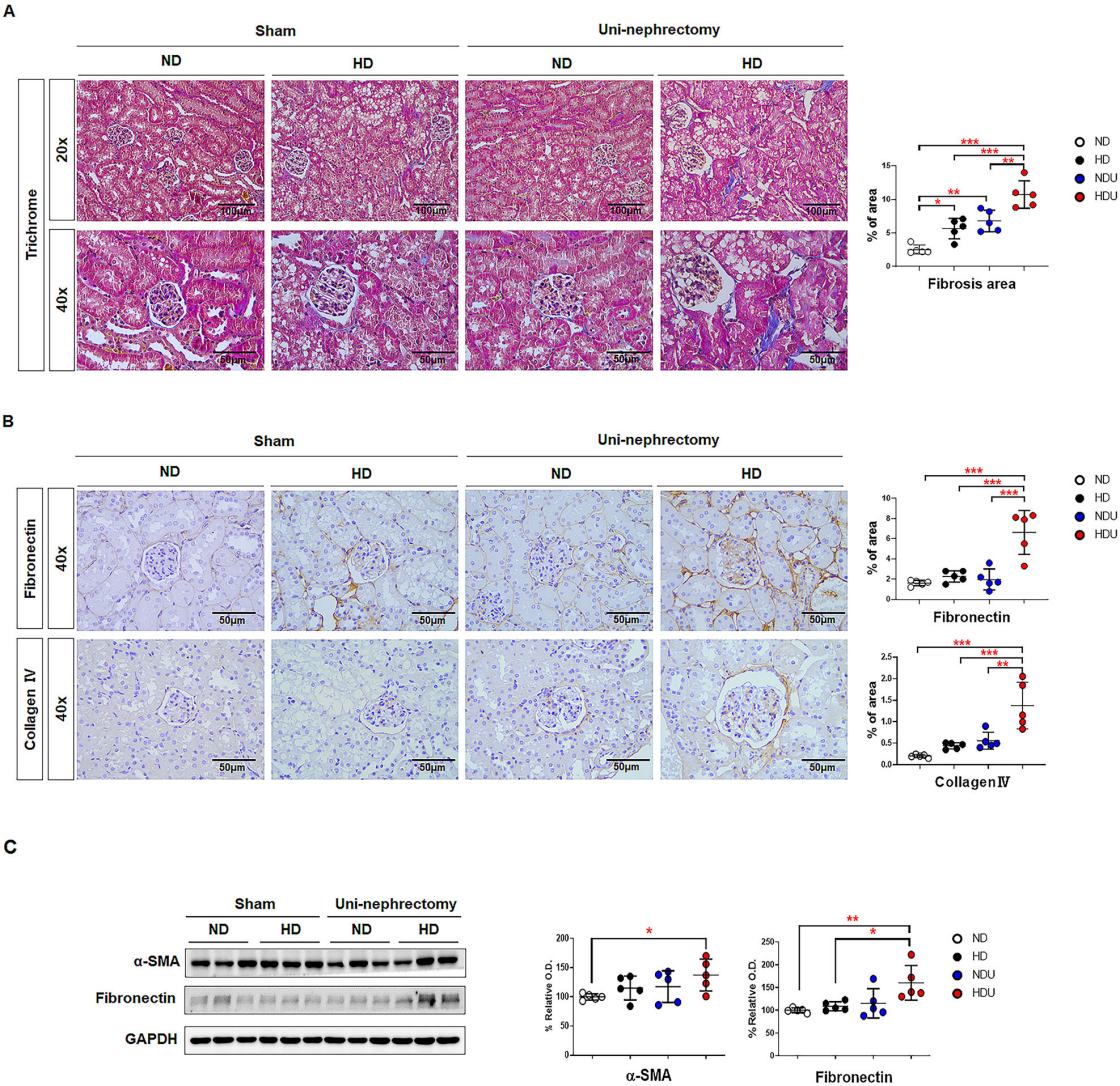
Confirmation of pathological damage, renal fibrosis due to changes in lipid metabolism in the unilateral renal injury model. **A** Representative histology of trichrome staining of the kidney (20×, 40×). Bar graph summarizes the percentage of the positive staining area. *n*  =  5 each group. **B** Immunohistochemistry for fibronectin and collagen IV (40×) of the kidney. Bar graph summarizes the percentage of the positive staining area. *n* = 5 each group. **C** Expression of fibrosis-associated proteins (α-SMA and fibronectin) in the kidney tissues was determined via Western blotting analysis. The band intensities were determined using densitometry with GAPDH ratios. Each bar represents the mean ± SEM. * *p* < 0.05, ** *p* < 0.01, *n* = 5 each group. ND sham mice fed with a normal diet, HD sham mice fed with a high-fat diet, NDU uninephrectomized mice fed with a normal diet, HDU uninephrectomized fed with a high-fat diet.

Trichrome staining showed fibrotic changes in the kidneys of the HDU group (Fig. 8A). A graph quantifying fibrosis also showed a significant increase in the HD and HDU groups compared to the ND group, with the HDU group exhibiting the most pronounced fibrosis. Kidney fibrosis markers, including fibronectin and collagen IV, were also increased in the interstitium and glomeruli in the HDU group (Fig. 8B). Further quantification confirmed elevated fibronectin expression in both the HD and HDU groups compared to the ND group, with the highest α-SMA expression observed in the HDU group (Fig. 8C), which is consistent with the expression changes observed in the histological analysis. This suggests that the high-fat diet induces progressive kidney fibrosis in the unilateral kidney model.

## Discussion

This study examined the impact of a high-fat diet on lipid metabolism in podocytes. Blood biochemistry showed that BUN and creatinine levels significantly increased in mice that underwent unilateral nephrectomy. Notably, the kidney weight of the HDU group increased owing to a greater glomerular size and a higher number of vacuolated tubule cells compared with those of the other groups. These findings suggest that high-fat-diet-induced lipid accumulation in podocytes may cause structural changes and impair renal function.

A high-fat diet is associated with increased cholesterol synthesis in the kidney and subsequent lipid accumulation in a unilateral kidney model [7]. Notably, the present study identified a specific increase in CE 20:4 levels in the podocytes of mice with a high-fat diet and unilateral kidney injury. In unilateral kidneys, the increase in CE 20:4 levels caused by a high-fat diet is supported by the presence of increased lipid droplets and vacuoles in podocytes. CE plays a role in cellular lipid storage and transport, and it is stored intracellularly as cytoplasmic lipid droplets_._ CE induces renal cell damage by increasing LCAT and ACAT levels and decreasing ABCA1 levels when diabetic nephropathy (DN) is fed a high-fat diet, leading to abnormal lipid metabolism [6]. The present study shows that lipid accumulation in podocytes might result in increased conversion to CE by ACAT1 rather than cholesterol and FFA synthesis. Normal lipid transport and degradation for maintaining cellular function is regulated partly by CD36, ABCA1, PPAR-γ, and LXRα [9, 10]. A previous study on lipid abnormalities in podocytes has reported increased lipid accumulation and damage caused by high CD36 levels and low ABCA1 levels [11]. The present study shows that the influx of CE 20:4 in CIHP-1 cells and in vitro cultured podocytes reduced the expression of ABCA1 and PPARα, leading to reduced lipid efflux.

Based on previous studies and our results, mitochondrial function and autophagy in podocytes were investigated to identify the mechanisms of CE20:4-induced kidney damage associated with a high-fat diet. In the kidney, the mitochondria respond rapidly to cellular damage and metabolic changes, such as nutritional and energy status [12]. Mitochondrial damage and division caused by cellular stress trigger cellular adaptation mechanisms that lead to the breakdown and autophagic clearance of mitochondria (mitophagy) or apoptosis [13]. The mitochondrial lipid oxidation process is extremely important in energy production [14]. Abnormalities in mitochondrial fission, fusion, and biogenesis, which represent mitochondrial function, can induce kidney damage in various kidney disease models such as DN and acute kidney injury [15, 16]. However, there is limited research on mitochondrial damage caused by the accumulation of specific lipid metabolites in podocytes, which perform important functions in kidney cells.

The present study shows that the high-fat diet-induced CE 20:4 accumulation in podocytes was associated with decreased expression of CPT1A and CPT2, which are key regulators of mitochondrial fatty acid oxidation. Additionally, mitochondrial fission increased, whereas mitochondrial activity and glycolytic function were reduced. These findings suggest that CE 20:4-induced mitochondrial damage may contribute to the dysfunction of podocytes. Interestingly, mitochondrial damage and biogenesis inhibition by CE 20:4 were associated with reduced levels of cardiolipin, NRF½, and PGC1α in CIHP-1 cells. The reduced levels of cardiolipin are closely linked to mitochondrial damage and have the potential to trigger mitophagy [17], which was also observed in our study. Although the present data suggest that lipid accumulation, particularly CE 20:4, impairs mitochondrial function, the possibility that pre-existing mitochondrial dysfunction contributes to lipid accumulation by reducing fatty acid β-oxidation cannot be excluded [18]. Further studies involving targeted manipulation of mitochondrial function, such as genetic or pharmacological approaches, are required to clarify the temporal relationship between these processes.

The mitophagy and autophagy pathways are interconnected in maintaining cellular homeostasis and responding to stress. In obesity-related metabolic disorders, the mitophagy pathway, particularly the PINK/Parkin-mediated mechanism, plays a crucial role in mitochondrial health [19]. Podocytes, which are terminally differentiated cells, use autophagy rather than cell division to minimize the intracellular buildup of defective DNA and other unwanted macromolecules [20]. However, their role in high-fat diet-induced podocyte injury of the unilateral kidney is not known. The present study shows that the expression of Beclin-1 and p62 increased with a high-fat diet in CIHP-1 cells and the unilateral kidney model. Meanwhile, the kidney tissues showed decreased expression of LC3A/B-I and increased expression of ATG5, suggesting an altered autophagy. These findings indicate that autophagy, which is essential for maintaining podocyte function, is dysregulated in response to high-fat diet-induced lipid accumulation, contributing to kidney dysfunction [21].

In our study, transcriptome analysis revealed significant upregulation of fibrotic markers with a high-fat diet (the HD and HDU groups). GO enrichment analyses of DEGs revealed a strong correlation between high-fat diet and fibrosis, particularly in cellular components associated with ECM organization and wound healing. In particular, genes such as *MMP12*, *MMP3*, *Col8a1*, and *Col27a1*, which are involved in ECM remodeling and fibrogenesis [22–24], were upregulated. These changes are consistent with the progression of fibrosis associated with CKD. In particular, MMP12 induces glomerular fibrogenesis and inflammation triggered by a high-fat diet [25]. The upregulation of MMP12 and MMP3 observed in the HDU group likely reflects a complex interplay of pro-inflammatory and pro-fibrotic signaling cascades in response to lipid accumulation and podocyte injury [25]. MMPs degrade ECM components that are components of the glomerular basement membrane [26]. MMPs contribute to the rupture of the basement membrane of glomeruli or Bowman’s capsule, resulting in macrophage infiltration into Bowman’s space, thereby promoting fibrosis of damaged tissue [27]. Future studies are needed to determine the pathophysiological mechanisms of MMP expression in the injured kidney and to determine whether regulation of MMP activity can alleviate renal fibrosis.

**Fig. 9:**
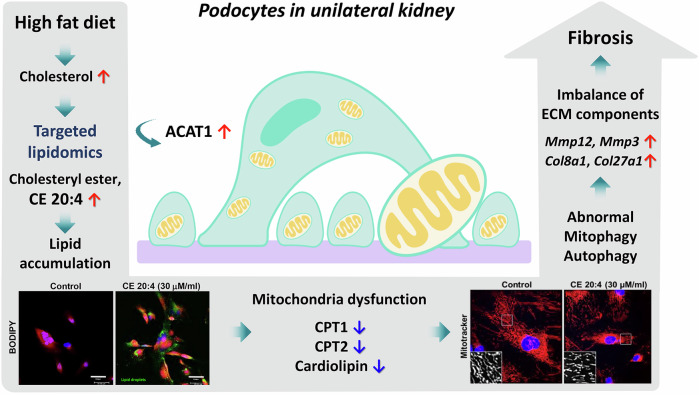
A summary of the results of the present study. High-fat diet induced mitochondrial damage and abnormal autophagy promote lipotoxicity in podocyte of uninephrectomized mice. Lipidomics analysis discovered cholesteryl ester 20:4, a lipid metabolite, that induces podocyte injury in unilateral kidney with high fat diet. It influences kidney damage and fibrosis.

Figure 9 summarizes these results. High-fat diets contribute to lipid imbalances in podocytes, with a particular focus on CE 20:4. Intracellular lipotoxicity with elevated CE 20:4 impairs mitochondrial function and induces abnormal autophagy activity. This promotes an imbalance between the production and degradation of ECM components, leading to renal function decline and fibrosis.

In conclusion, our study demonstrated that a high-fat diet in a single kidney model leads to alterations in renal lipid metabolism, particularly the accumulation of CE 20:4 in podocytes. This accumulation is associated with mitochondrial dysfunction, impaired autophagy, and kidney fibrosis. Further research, including the temporal relationship between lipid accumulation and mitochondrial dysfunction, as well as the role of mitophagy and autophagy, may help to better understand and potentially inhibit kidney damage associated with obesity and high-fat diets.

## Material and methods

Methodologies, including animals and cell culture experiments, biochemistry and histology, transmission electron microscopy (TEM), transcriptome sequencing analysis, gene ontology enrichment analysis, podocytes cell isolation, lipid extraction [28], targeted lipidomics analysis [29, 30], cholesterol and free fatty acids (FFA) analysis [31], cardiolipin analysis [32, 33], Oil Red O staining, immunofluorescence assay, mitotracker, mitochondrial oxygen consumption rate (OCR) and extracellular acidification rate (ECAR) measurements, ATPase activity measurement, western blotting, and statistical analysis are detailed in the Supplementary Methods.

### Experiment schedules

As shown in Supplementary Fig. S1, the experimental outline of this study is divided into in vivo and in vitro experiments. The primary antibodies used are listed in Table 1.

**Table 1:** Antibodies used in the current study.

Primary antibody information					
Protein name	Abbreviation	Host	Application	Cat. no.	Source
Acetyl-CoA acetyltransferase 1	ACAT1	Rabbit	1:1000	ab168342	Abcam
Lecithin cholesterol acyl transferase	LCAT	Mouse	1:500	sc-398361	Santa Cruz Biotechnology
Peroxisome proliferator-activated receptor gamma	PPARγ	Rabbit	1:1000	2435s	Cell Signaling Technology
Liver X receptor alpha	LXRα	Rabbit	1:1000	ab176323	Abcam
Cluster of differentiation 36	CD36	Rabbit	1:1000	ab133625	Abcam
Fatty acid transport protein 2	FATP2	Mouse	1:1000	sc-393906	Santa Cruz Biotechnology
ATP binding cassette subfamily A member 1	ABCA1	Mouse	1:1000	ab18180	Abcam
Peroxisome proliferator-activated receptor alpha	PPARα	Mouse	1:500	SC-398394	Santa Cruz Biotechnology
Carnitine palmitoyltransferase 1A	CPT1A	Mouse	1:1000	ab128568	Abcam
Carnitine palmitoyltransferase 2	CPT2	Rabbit	1:1000	ab181114	Abcam
Acyl-CoA oxidase 1	ACox1	Rabbit	1:1000	ab184032-1	Abcam
pyruvate dehydrogenase kinase 4	PDK4	Mouse	1:500	ab110336	Abcam
Dynamin-related protein 1	DRP1	Rabbit	1:1000	ab184247-1	Abcam
Peroxisome proliferator-activated receptor-γ coactivator 1-alpha	PGC1α	Goat	1:500	ab106814	Abcam
Nuclear respiratory factor 1 and 2	NRF½	Rabbit	1:500	ab175932	Abcam
Beclin-1		Rabbit	1:1000	3738s	Cell Signaling Technology
Autophagy related 5	Atg5	Rabbit	1:1000	2630s	Cell Signaling Technology
Microtubule-associated protein 1 light chain 3A and B	LC3A/B	Rabbit	1:1000	12741s	Cell Signaling Technology
Sequestosome 1 and p62	SQSTM1/P62	Rabbit	1:1000	5114s	Cell Signaling Technology
Tumor necrosis factor-alpha	TNFα	Rabbit	1:1000	ab6671	Abcam
NOD-, LRR- and pyrin domain-containing protein 3	NLRP3	Rabbit	1:1000	13158s	Cell Signaling Technology
Interleukin 1 beta	IL-1β	Mouse	1:1000	12242s	Cell Signaling Technology
Fibronectin		Rabbit	1:1000	ab2413	Abcam
Alpha-smooth muscle actin	α-SMA	Mouse	1:500	A2547	Sigma-Aldrich
Collagen-IV		Rabbit	1:1000	ab6586	Abcam
B-cell lymphoma 2	BCL-2	Rabbit	1:1000	ab196495	Abcam
Sterol regulatory element binding transcription factor 1	SREBP1	Mouse	1:500	SC-365513	Santa Cruz Biotechnology
Sterol regulatory element binding transcription factor 2	SREBP2	Mouse	1:500	SC-13552	Santa Cruz Biotechnology
Acetyl-CoA carboxylase	ACC	Rabbit	1:1000	3676S	Cell Signaling Technology
Phospho-acetyl-CoA carboxylase	p-ACC	Rabbit	1:1000	3661s	Cell Signaling Technology
Fatty acid synthase	FAS	Rabbit	1:1000	3180s	Cell Signaling Technology
3-Hydroxy-3-methylglutaryl-CoA reductase	HMGCR	Mouse	1:1000	ab174830-1	Abcam
Glyceraldehyde-3-phosphate dehydrogenase	GAPDH	Rabbit	1:5000	2118	Cell Signaling Technology

## Supplementary information

Supplementary material

Western blot raw band

## Data Availability

The lipidomic analysis data of this study can be found in the Korea BioData Station, accession number PRJKA628271 (https://kbds.re.kr/). The transcriptomic analysis data of this study can be found in the NCBI Sequencing Read Archive database, accession number PRJNA1065123 (http://www.ncbi.nlm.nih.gov/sra/).
