# Supplementary material for: High-fat diet promotes lipotoxicity in the podocytes of uninephrectomized mice: a targeted lipidomics and kidney podocyte-specific analysis
Source: Cell Death Discov. 2025 Apr 23;11:193. doi: 10.1038/s41420-025-02419-7 (PMC12019177; doi:10.1038/s41420-025-02419-7)
Supplement: Supplementary file 1 — Supplementary material [file 41420_2025_2419_MOESM1_ESM.docx]

**SUPPLEMENTARY MATERIAL**

**Supplementary Methods**

**In vivo analysis**

***Animals, diets, and uninephrectomy surgery***

Five-week-old male C57BL/6 mice weighing 16–17 g were obtained from Hyochang Science (Dague, South Korea). Mice were housed with free access to chow and water and were kept in specific pathogen free (SPF) units with a 12-light/dark cycle. All animal experiments were approved by the Animal Care and Use Committee of Kyungpook National University (KNU-2021-0032). At 13 weeks, the mice were fed with normal diet (ND, standard diet) containing 20.3% protein, 5% fat, and 66% carbohydrate. After uninephrectomy (UN), some mice were fed with high-fat diet (HD, diet D12451; rodent diet with 45 Kcal% fat). Randomized mice underwent UN at 1 week. The mice were divided into the ND (n = 6), HD (n = 6), normal diet and uninephrectomy (NDU) (n = 12), and high-fat diet and uninephrectomy (HDU) (n = 12) groups. After the mice received anesthesia with 3%–5% isoflurane, their left kidney was surgically removed. At 13 weeks after surgery, all mice were sacrificed, and the kidneys were collected for analysis. Half of the kidneys were used for molecular analysis and the other half for histological analysis.

***Biochemistry***

All mice were weighed weekly and euthanized via cardiac puncture under anesthesia at 13 weeks after UN. The kidney weight was measured after removing the Gerota’s fascia on the day of sacrifice. Blood samples and the kidneys were collected for analysis. After centrifugation of each blood sample, the serum was used for biochemical analysis. The blood urea nitrogen (BUN), creatinine (Cr), and total cholesterol levels were assessed by GC Labs (Yongin, South Korea) using the Cobas 8000 modular analyzer system (Roche, Germany).

***Histology***

The kidney tissues collected from each experimental group were fixed with 4% paraformaldehyde (pH 7.4) and embedded in paraffin. Two-micrometer tissue sections were incubated with collagen Ⅳ antibody (1:100, ab6586, abcam, the USA), fibronectin antibody (1:100, ab2413, Abcam, the USA), and α-SMA antibody (1:100, A2547, Sigma-Aldrich, the USA) overnight at 4°C. Horseradish-peroxidase-conjugated polyclonal goat anti-rabbit immunoglobulin (Dako, Glostrup, Denmark) was used as the secondary antibody. The DAB Peroxidase Substrate Kit (Vector Laboratories, Burlingame, CA) was utilized to visualize the stained tissues. The sections were prepared and stained with PAS using standard protocols to determine histological changes. The glomerular size (μm^2^) was measured as the length of each glomerular using Image J (NIH) in more than nine randomly selected fields in the cortex sections. The number of vacuolated tubular cells was counted from more than 10 random sections or 10 high-power fields from the outer medulla of each sample. Then, the average were obtained.

***Transmission* *electron microscopy***

The kidney tissue samples were fixed overnight in 2.5% glutaraldehyde at 4°C for 12 h and cut into 1-mm 3-sized blocks. The cut tissues were washed with 0.1 M phosphate buffer (pH 7.2) and post fixed with 2% osmium tetroxide for 90 min. After washing three times with 0.1 M phosphate buffer (pH 7.2) for 10 min each time, the samples were dehydrated via a graded series of 50%–100% ethanol and 100% propylene oxide, and were infiltrated in 1:1, 1:2, and 1:3 mixtures of propylene oxide:epon for 1 h. The samples were incubated in 100% EPON for 8 h and cured at 35°C and 45°C for 12 h at each temperature, and were additionally hardened at 60°C for 48 h. After trimming, the ultrathin (60 nm) sections were double-stained with 2% uranyl acetate for 25 min and 1% lead citrate for 15 min. The sections were visualized at 75 kV with the H7000 transmission electron microscope (Hitachi, Tokyo, Japan). A minimum of three different grids in each animal group was examined. All glomeruli in a grid were examined at a magnification of x 3000 to identify podocytes and endothelial and mesangial cells. The mitochondria structure of podocytes and endothelial cells was then examined at a higher magnification. Osmium staining for electron microscopic analysis was done for lipid droplets accumulated in the kidney.

***Transcriptome sequencing analysis***

After kidney biopsy were obtained, kidney biopsy were storage in RNAlater solution (Ambion) and then frozen in −80°C. One kidney per mouse was used to generate one RNA sample, and three sets of RNA samples (three mice per group; ND, NDU, HD, and HDU groups) were processed for RNA sequencing. RNA extraction, library preparation, cluster generation, and sequencing were performed by Macrogen Inc. (Seoul, South Korea). RNA samples for sequencing were prepared using the TruSeq Stranded mRNA Library Prep Kit (Illumina platform) according to the manufacturer’s instructions.

The raw data for each sample and the trimmed reads that went through the pre-processing process were compared to the total data amount and Q30 (phred score, base quality ≥ 30) values. Trimmed reads that were processed for spliced read mapping through the Bowtie2 aligner were mapped to the known Mus musculus genome (mm10) using the HISAT2 program. After read mapping, transcript assembly was performed using the StringTie program. Consequently, expression profile values were obtained for each sample for known transcripts, and read count, fragment per kilobase of transcript per mil-lion mapped reads (FPKM), and transcripts per kilobase million values were organized based on transcript/gene. Statistical analysis used fold change and the nbinomWaldTest in DESeq2 for each comparative combination. Using this value, differentially expressed gene (DEG) analysis was performed using DESeq2 for four comparison combinations (ND, NDU, HD, and HDU). Fold-changes (FCs) in the gene expression values were calculated, and FC > 2 and *P*-value < 0.05 were considered the cutoff values for identifying DEGs.

***Gene Ontology (GO) enrichment analysis***

GO is a structured, controlled vocabulary to unify the representation of genes and classify gene functions. The enriched GO terms included three non-overlapping ontologies, including biological process (BP), cellular component (CC), and molecular function (MF). For significant DEGs, gProfiler (<https://biit.cs.ut.ee/gprofiler/orth>https://biit.cs.ut.ee/gprofiler/orth, accessed on 16 August 2022) was used. All analyses were performed by Macrogen Inc. (Seoul, South Korea).

**Lipodomics analysis**

***Podocyte cell isolation in mouse kidneys***

Initially, the kidney was decapsulated, chopped, and then incubated at 37°C for 40 min with collagenase (2 mg/mL) (Sigma-Aldrich, St. Louis, the USA) in RPMI-1640 medium containing penicillin, streptomycin, and 5% fetal bovine serum. Then, the specimens were passed through a cell strainer (100 µm) (BD Biosciences). To remove the red blood cells, the cell suspension was centrifuged at 300 g for 5 min at 4°C. Further digestion was performed in the medium comprising collagenase (0.5 mg/mL), dispase II, and 0.075% trypsin with mild rotation (37°C, 20 min). To incompletely remove separated renal tissues, the samples were passed through a cell strainer (25 μm) and centrifuged at 300 g for 5 min. For the purification of podocytes, after washing with bead buffer (0.5% BSA, 0.5 M EDTA in phosphate buffered saline), renal single cells (1 × 10^7^) were incubated with 10 μg of IgG anti-nephrin monoclonal antibody (ab136894, Abcam, the USA) for 10 min at 4°C. Then, 20 µl of IgG-mouse MicroBeads (Miltenyi) were added, and the cells were incubated for 15 min at 4°C. To remove unbound IgG beads, the cells were washed with bead buffer. Using anti-nephrin antibody (ab136894, Abcam, the USA), the nephrin-positive cells were isolated using second MACS.

***Lipid extraction***

All lipid extractions using kidney tissues and epithelial and podocyte cells were performed using the Matyash method after slight modification Next, 75% ice-cold methanol (400 µL) containing 0.1% butylated hydroxytoluene was added in the kidney tissues, epithelial cells, and podocytes. After homogenizing kidney tissues, epithelial cells and podocytes were removed using stainless steel beads and TissueLyser (QIAGEN, Germany’s Helden). After adding 1 mL of methyl-*tert*-butyl ether with 0.1% butylated hydroxytoluene, the samples were shaken at room temperature for 1 h. In total, 250 µL of water was added and vortexed for 10 min. Next, phase separation was performed via centrifugation at 14,000 g for 15 min at 4°C. For targeted lipidomics analysis, the upper (550 μL) and lower (275 μL) phases were pooled and dried using N_2_ purge. To analyze free fatty acids and cholesterol, the upper (110 μL) and lower (555 μL) phases were pooled and dried using N_2_ purge.

***Targeted lipidomics analysis***

The dried lipid extracts were reconstituted in 100 μL of chloroform/methanol (1/9, *v/v*) containing internal standard mixture (IS) prior to lipidomics analysis. The levels of lipids (phosphatidylcholine (PC), lysoPC, plasmenyl PC, phosphatidylethanolamine (PE), lysoPE, plasmenyl PE, monoacylglycerol, diacylglycerol, triacylglycerol, acylcarnitine, ceramide, sphingomyelin, and cholesteryl ester) were analyzed using liquid chromatography-triple quadrupole mass spectrometry (LC-MS/MS, Mass Spectrometry Based Convergence Research Institute, Kyungpook National University, Shimadzu LCMS 8060, Shimadzu Corporation, Kyoto, Japan), based on a previously reported method with some modifications.^9, 10^ Each lipid quantitation was performed via selected reaction monitoring (SRM), and SRM transition ions for each lipid class and IS are described in our previously published paper.^10^ Metabolite expression normalization, heatmap analysis, and statistical analysis were performed using Multi-Experiment Viewer software (version 4.9.0).

***Analysis of cholesterol and free fatty acids***

Cholesterol and free fatty acid (FFA) levels were analyzed using gas chromatography-mass spectrometry, as reported in a previous study. Briefly, cholesterol and FFAs were derivatized via incubation with *N*-tert-butyldimethylsilyl-*N*-methyltrifluoroacetamide with ammonium iodide in pyridine at 80°C for 5 min. Reaction mixtures were dried, reconstituted with *n*-hexane, and then injected into gas chromatography-mass spectrometry (QP2100 Ultra, Shimadzu Corporation).

***Cardiolipin analysis***

The upper phase of lipid extracts (550 μL) was dried and reconstituted with 100 μL of methanol. The upper layer (1 mL) of methyl-*tert*-butyl ether/methanol/water (10/3/2.5, *v/v/v*) and 2 M trimethylsilyldiazomethane (100 μL) in hexane were added into the reconstituted sample. After mixing for 30 min, glacial acetic acid (5 μL) was added to quench the reaction. The sample was dried under N_2_ purge, reconstituted with 100 μL of chloroform/methanol (1/9, *v/v*), and analyzed via LC-MS/MS with the Kinetex C18 column (100 × 2.1 mm, Phenomenex, Torrance, CA, the USA). The LC-MS/MS operating conditions were similar to the conditions used in targeted lipidomics analysis. Each cardiolipin (CL) quantitation was performed using SRM. Supplementary Table 1 shows the SRM transitions and collision energies of each CL and IS (CL 57:4 (14:1/14:1/14:1/15:1). The metabolite expression normalization, heat map analysis and statisical analysis were performed using Multi-Experiment Viewer software (MeV) (version 4.9.0).

**In vitro analysis**

***Cell culture and treatments***

The Human Podocyte Cell Line (CIHP-1) was purchased from Ximbio (London, the UK). The CIHP-1 cells were cultured on collagen I-coated dishes. The cells were cultured in RPMI-1640 supplemented with 10% fetal bovine serum and antibiotic-antimycotic (100 U/mL of penicillin, 100 µg/mL of streptomycin, and Amphotericin B) at two different temperatures. After the proliferation of podocytes at 33°C, cells were cultured for 14 days at 37°C for the induction of differentiation. The cholesteryl ester (CE) 20:4 (Cholesteryl Arachidonate, 22595, Cayman Chemical, Michigan, the USA) was dissolved in dimethyl sulfoxide (DMSO) at high concentration (10 mM). The stock solution was then diluted in cell culture medium to obtain the desired final concentration. Care should be taken to ensure that the final concentration of the organic solvent does not exceed 0.1%. To ensure uniform dispersion of CE 20:4, the medium was preheated to 37°C to facilitate micelle formation. The cultured human podocytes were starved under serum-deprived conditions for 24 h and treated with cholesteryl ester (CE) 20:4 (Cholesteryl Arachidonate, 22595, Cayman Chemical, Michigan, the USA) for 24 h.

***Oil Red O staining***

The CIHP-1 cells were rinsed in PBS and then incubated in 4% paraformaldehyde for 30 min at 4℃. Cells were washed with ddH_2_O twice and with 60% isopropanol for 5 min at room temperature. After the cells dried up completely, they were stained for 30 min in Oil Red O solution at room temperature. The Oil Red O solution was removed, and the cells were immediately washed four times with ddH_2_O. Nuclear staining was performed with hematoxylin.

***Immunofluorescence assay***

The CIHP-1 cells were plated on the coverslips at the appropriate density. The adherent cells were fixed with 4% paraformaldehyde for 10 min on ice. Permeabilization was achieved by treating cells with PBS containing 0.1% Triton X-100 for 10 minutes at room temperature to allow anti-Tom20 antibodies to access the mitochondrial protein Tom20. A blocking solution such as 10% bovine serum albumin (BSA) was then applied for 1 h at room temperature to prevent nonspecific antibody binding. The cells were then incubated with Tom20 Rabbit/IgG Polyclonal Antibody (1:200, 11802-1-AP, Proteintech, the USA) overnight at 4°C. The cells were incubated with Alexa Fluor® 488-labeled gout anti-rabbit immunoglobulin G (H + L) (1:200, A-11008, Invitrogen, the USA) for 60 min, and then with Alexa594-phalloidin (A12381, Invitrogen, USA) diluted in 1% BSA-PBS for 30 min at a final concentration of 5 U/mL. An antifluorescence quenching agent containing 4',6-diamidino-2-phenylindole was applied to the coverslips. Based on the lipid droplet stain protocol, BODIPY 493/503 dye was used. The cells were incubated for 30 min at room temperature with the staning solution diluted with DAPI (1:5000), 2 µg/mL of BODIPY 493/503 (1:500, D3922, Invitrogen, the USA), and HCS CellMask Deep Red (1:80000, H32712, Invitrogen, the USA) in 1x PBS. All microscopic images were recorded using a confocal or automatic microscope (Olympus, Japan). Colocalization (%) and mitochondrial length (> 10 µm considered as elongated and < 10 µm as fragmented) were quantified using the Image J software with Fiji plugin (NIH).

***Mitotracker***

The CIHP-1 cells were plated on black well, clear-bottom, 96-well plate (655096, Greiner Bio-One, Thaliland). Before imaging, the cells were prestained with NucBlue (R37605; Thermo Fisher Scientific) and MitoTracker Deep Red (M22426; Thermo Fisher Scientific), and washed. Then, the medium was washed with fetal bovine serum free-RPMI media. The cell images were captured and tracked using the ImageXpress Micro Confocal (20×, Molecular Devices, the USA).

***Mitochondrial oxygen consumption rate and extracellular acidification rate measurement***

The oxygen consumption rate (OCR) and extracellular acidification rate (ECAR) of the cells were seeded on the Seahorse XF 96-well plate overnight. Before measurement, the cells were washed and equilibrated for 1 h at 37°C with the XF base medium (102353-100; Agilent technologies) supplemented with 1X GlutaMAX (35050; Gibco), 1 mM of sodium pyruvate (S8636; Sigma-Aldrich), and 25 mM of glucose (G7528; Sigma-Aldrich) (pH 7.4). Then, the pre-hydrated sensor cartridge loaded with the mitochondrial inhibitors to achieve a final concentration of 1 μM of oligomycin (75351; Sigma-Aldrich), 1 μM of FCCP (C2920; Sigma-Aldrich), and 0.5 μM of rotenone (R8875; SigmaAldrich) + 0.5 μM of antimycin A (A8674; Sigma-Aldrich) was placed on the XF 96-well plate. Then, OCR was measured before and after the sequential injection of mitochondrial inhibitors. For ECAR measurement, the cells were washed, and the medium was replaced with glucose or pyruvate-free XF base medium supplemented with 1 mM of glutamine (G8540; Sigma-Aldrich) for 1 h before the assay. Then, the cells loaded with sensor cartridge containing glucose, oligomycin (75351; Sigma-Aldrich), and 2-deoxyglucose (D6134; Sigma-Aldrich) were injected to a final concentration of of 10 mM, 2 μM, and 50 mM, respectively. OCR and ECAR were measured using the Seahorse XFe 96 analyzer (Agilent technologies) and analyzed using the Wave 2.6.0 software after normalization with the total cell number.

***Western blotting***

After treatment with various agents, cells and tissues were lysed in RIPA buffer (50 mmol/L Tris-HCl, 150 mmol/L NaCl, 1% NP-40, 0.5% sodium deoxycholate, and 0.1% sodium dodecyl sulfate) and Protease Inhibitor Cocktail Set III (Calbiochem, Darmstadt, Germany). The lysates were centrifuged at 12000 × *g* for 15 min, and the protein concentration was measured using the Bradford’s method. The total protein (20 µg from each sample) was separated on 8-12% SDS-polyacrylamide gel and transferred to a nitrocellulose membrane. The membrane was blocked with 10% nonfat dry milk in 10 mmol/L Tris-buffered saline with 0.1% Tween 20 (TBS-T) for 1 h. Next, it was incubated with diluted primary antibodies in TBS-T overnight at 4°C. A horseradish-peroxidase-conjugated polyclonal goat anti-rabbit immunoglobulin or goat anti-mouse immunoglobulin (Dako, Glostrup, Denmark) was used as the secondary antibody for Western blotting. Positive immunoreactive bands were quantified via densitometry and were compared with the glyceraldehyde-3-phosphate dehydrogenase expression. The expression levels were assessed using the Scion Image software (Scion, Frederick, MD, the USA). Supplementary Table 2 depicts the primary antibodies used in this study.

***Statistical analysis***

Analysis data are presented as mean ± standard error of the mean (SEM). Statistical analyses were performed by one-way analysis of variance (ANOVA) with Tukey’s post-hoc analysis for normally distributed data or Kruskal–Wallis test with Bonferroni correction for non-normally distributed data. The Shapiro-Wilk test was used to evaluate whether the data were normally distributed. All analysis were performed using R software (R Foundation for Statistical Computing, Vienna, Austria; [www.r-project.org](http://www.r-project.org)) A *p*-value less than 0.05 was considered statistically significant.

All lipidomics analysis data exported by Shimadzu LabSolutions LC-MS software were processed in Excel. In each group, replicates were used for data processing with a mean ± standard deviation (SD). Processed Excel data results are exported to SIMCA-P + software (version 14.1, Umetrics, Umea, Sweden) for multivariate statistical. To identify the stability and reproducibility of the instrument, principal component analysis (PCA) was performed. Partial least squares-discriminant analysis (PLS-DA) was performed to examine whether each group formed a cluster. Hotelling’s T2 test was used to exclude outliers from the 95% confidence region. GraphPad Prism 7.0 (Graph Pad Software, San Diego, CA the USA) was used for graph and statistical analysis.

**Supplementary Dataset**

**Supplementary Table S1. LC/MS.MS used in the current study.**

| **No.** | **Class** | **Species** | **Adduct ion** | **Q1 (*m/z*)** | **Q3 (*m/z*)** | **CE (eV)** | **Retention time (min)** |
| --- | --- | --- | --- | --- | --- | --- | --- |
| 1 | CL* | 57:4(14:1/14:1/14:1/15:1) | [M+NH_4_]^+^ | 1292.9 | 491.4 | 35 | 6.59 |
| 2 | CL | 66:5(16:1/16:1/16:1/18:2) | [M+NH_4_]^+^ | 1417.0 | 547.5 | 35 | 7.75 |
| 3 | CL | 66:5(16:1/14:1/18:2/18:1) | [M+NH_4_]^+^ | 1417.0 | 519.5 | 35 | 7.77 |
| 4 | CL | 68:6(18:2/18:2/16:1/16:1) | [M+NH_4_]^+^ | 1443.0 | 599.5 | 35 | 7.84 |
| 5 | CL | 68:5(16:1/16:1/18:2/18:1) | [M+NH_4_]^+^ | 1445.0 | 547.5 | 35 | 8.12 |
| 6 | CL | 68:5(18:2/16:1/18:1/16:1) | [M+NH_4_]^+^ | 1445.0 | 573.5 | 35 | 8.12 |
| 7 | CL | 68:4(18:2/16:0/18:2/16:0) | [M+NH_4_]^+^ | 1447.1 | 575.5 | 35 | 8.43 |
| 8 | CL | 68:4(16:1/16:0/18:2/18:1) | [M+NH_4_]^+^ | 1447.1 | 601.5 | 35 | 8.47 |
| 9 | CL | 68:3(18:1/16:1/18:1/16:0) | [M+NH_4_]^+^ | 1449.1 | 575.5 | 35 | 8.83 |
| 10 | CL | 68:2(18:1/16:0/18:1/16:0) | [M+NH_4_]^+^ | 1451.1 | 577.5 | 35 | 9.29 |
| 11 | CL | 70:9(18:2/18:2/18:3/16:2) | [M+NH_4_]^+^ | 1465.0 | 599.5 | 35 | 6.89 |
| 12 | CL | 70:8(18:3/18:2/18:2/16:1) | [M+NH_4_]^+^ | 1467.0 | 597.5 | 35 | 7.77 |
| 13 | CL | 70:7(18:2/18:2/18:2/16:1) | [M+NH_4_]^+^ | 1469.0 | 599.5 | 35 | 7.92 |
| 14 | CL | 70:6(18:2/18:2/18:2/16:0) | [M+NH_4_]^+^ | 1471.1 | 599.5 | 35 | 8.22 |
| 15 | CL | 70:6(18:2/18:1/18:2/16:1) | [M+NH_4_]^+^ | 1471.1 | 601.5 | 35 | 8.21 |
| 16 | CL | 70:5(18:2/18:1/18:2/16:0) | [M+NH_4_]^+^ | 1473.1 | 601.5 | 35 | 8.53 |
| 17 | CL | 70:4(18:2/18:1/18:1/16:0) | [M+NH_4_]^+^ | 1475.1 | 601.5 | 35 | 8.95 |
| 18 | CL | 70:3(18:1/18:1/18:1/16:0) | [M+NH_4_]^+^ | 1477.1 | 603.6 | 35 | 9.35 |
| 19 | CL | 72:9(18:2/18:2/18:2/18:3) | [M+NH_4_]^+^ | 1493.0 | 599.5 | 35 | 7.86 |
| 20 | CL | 72:8(18:2/18:2/18:2/18:2) | [M+NH_4_]^+^ | 1495.1 | 599.5 | 35 | 8.01 |
| 21 | CL | 72:7(18:2/18:1/18:2/18:2) | [M+NH_4_]^+^ | 1497.1 | 599.5 | 35 | 8.32 |
| 22 | CL | 72:6(18:1/18:1/18:2/18:2) | [M+NH_4_]^+^ | 1499.1 | 599.5 | 35 | 8.66 |
| 23 | CL | 72:6(18:2/18:1/18:2/18:1) | [M+NH_4_]^+^ | 1499.1 | 601.5 | 35 | 8.65 |
| 24 | CL | 72:5(18:1/18:1/18:1/18:2) | [M+NH_4_]^+^ | 1501.1 | 601.5 | 35 | 9.02 |
| 25 | CL | 72:4(18:1/18:1/18:1/18:1) | [M+NH_4_]^+^ | 1503.1 | 603.6 | 35 | 9.47 |
| 26 | CL | 74:10(18:2/18:2/18:2/20:4) | [M+NH_4_]^+^ | 1519.1 | 599.5 | 35 | 7.96 |
| 27 | CL | 74:9(18:2/18:2/18:1/20:4) | [M+NH_4_]^+^ | 1521.1 | 599.5 | 35 | 8.17 |
| 28 | CL | 74:8(18:2/18:2/18:0/20:4) | [M+NH_4_]^+^ | 1523.1 | 599.5 | 35 | 8.45 |
| 29 | CL | 74:8(18:1/18:2/18:1/20:4) | [M+NH_4_]^+^ | 1523.1 | 601.5 | 35 | 8.50 |
| 30 | CL | 74:7(18:1/18:2/18:0/20:4) | [M+NH_4_]^+^ | 1525.1 | 601.5 | 35 | 8.79 |
| 31 | CL | 74:6(18:1/18:2/18:0/20:3) | [M+NH_4_]^+^ | 1527.1 | 601.5 | 35 | 9.15 |
| 32 | CL | 76:11(18:1/18:2/20:4/20:4) | [M+NH_4_]^+^ | 1545.1 | 601.5 | 35 | 8.15 |
| 33 | CL | 76:11(18:2/18:2/20:3/20:4) | [M+NH_4_]^+^ | 1545.1 | 599.5 | 35 | 8.03 |
| 34 | CL | 76:11(18:2/20:4/18:1/20:4) | [M+NH_4_]^+^ | 1545.1 | 623.5 | 35 | 8.16 |
| 35 | CL | 76:10(18:1/20:4/18:1/20:4) | [M+NH_4_]^+^ | 1547.1 | 625.5 | 35 | 8.32 |
| 36 | CL | 76:10(18:1/18:2/20:3/20:4) | [M+NH_4_]^+^ | 1547.1 | 601.5 | 35 | 8.34 |
| 37 | CL | 76:10(18:2/18:2/20:3/20:3) | [M+NH_4_]^+^ | 1547.1 | 599.5 | 35 | 8.28 |
| 38 | CL | 76:9(18:1/20:4/18:0/20:4) | [M+NH_4_]^+^ | 1549.1 | 625.5 | 35 | 8.63 |
| * IS, internal standard | | | | | | | |

**
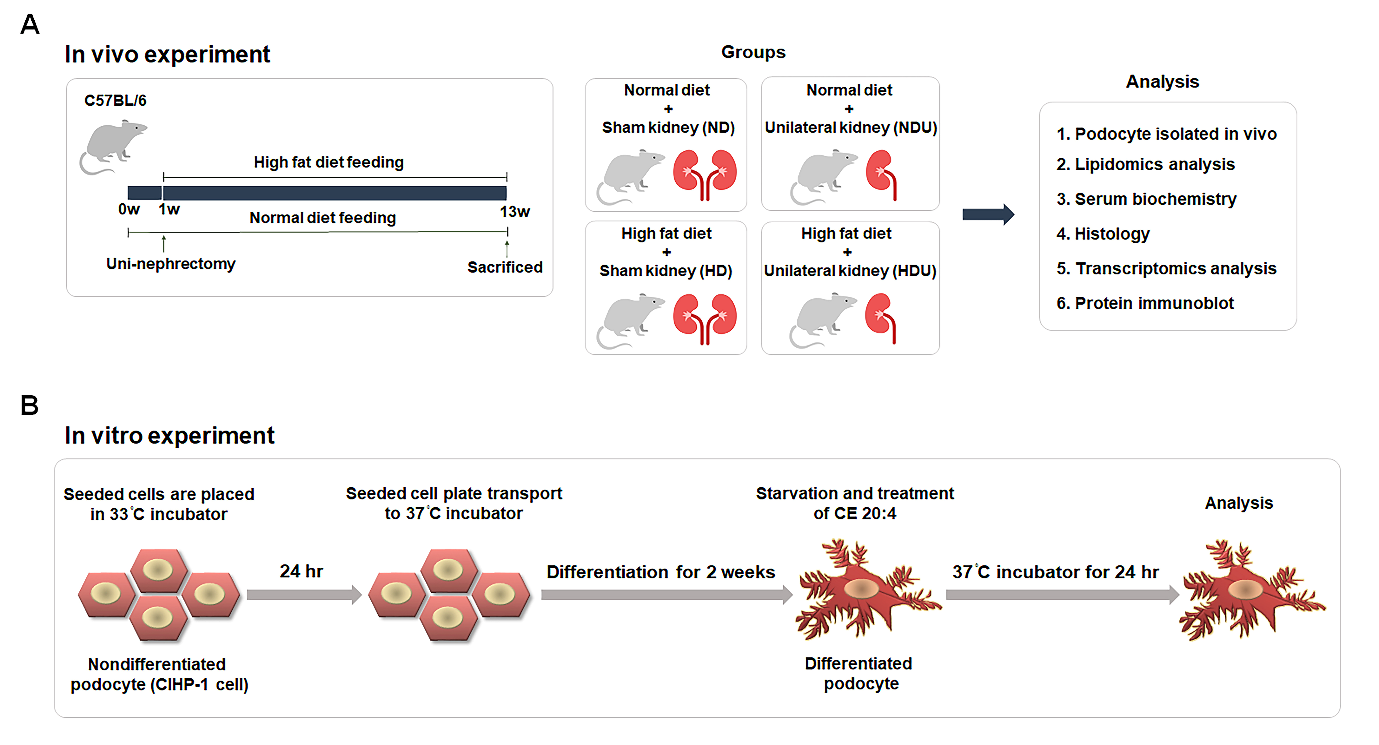
**

**Supplementary Figure S1. Experimental schedul**e**. (A)** In vivo experimental schedule. **(B)** In vitro experimental schedule.


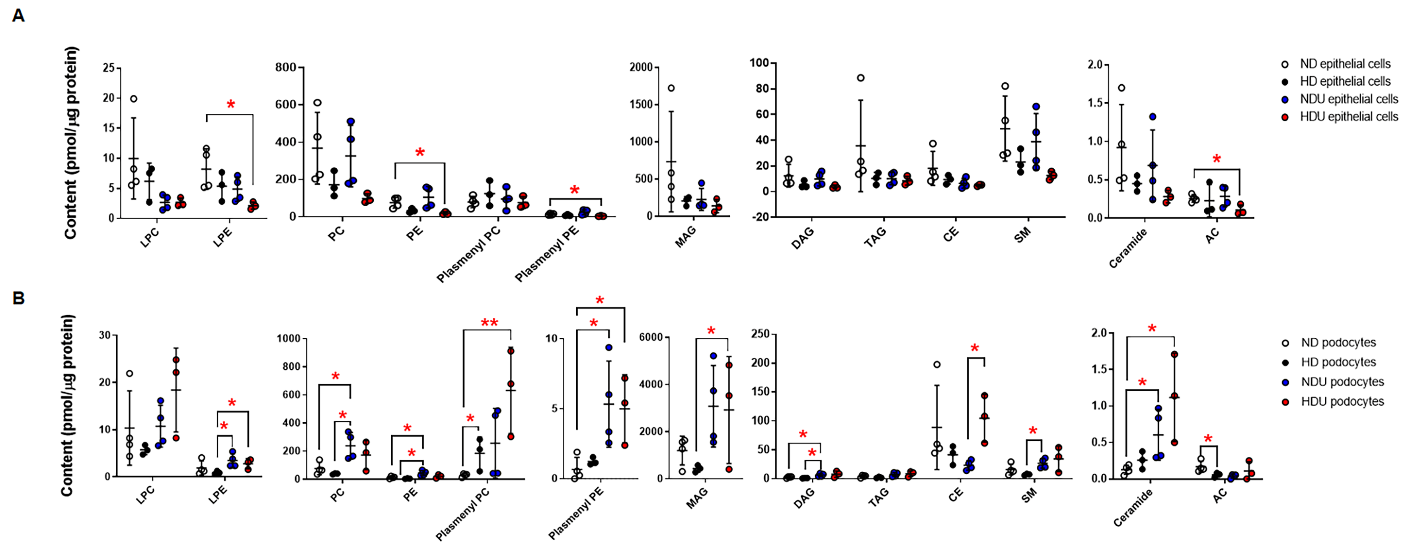


**Supplementary Figure S2. Overview of the effects of high-fat diet on (A) kidney epithelial cells and (B) podocyte lipidomes in uni-nepherectomized mice**. The total lipid contents are expressed as the sum of contents of all identified lipids in samples. The data are shown as mean ± standard deviation (SD) (*n* = 3–4). ND: sham mice fed a normal diet; HD: sham mice fed a high-fat diet; NDU: uni-nepherectomized mice fed a normal diet; HDU: uni-nepherectomized mice fed a high-fat diet.* *p* < 0.05, ** *p* < 0.01.
