# Supplementary material for: High-fat diet promotes lipotoxicity in the podocytes of uninephrectomized mice: a targeted lipidomics and kidney podocyte-specific analysis
Source: Cell Death Discov. 2025 Apr 23;11:193. doi: 10.1038/s41420-025-02419-7 (PMC12019177; doi:10.1038/s41420-025-02419-7)

Figure 3. Lipid Accumulation and Changes in lipid metabolism factors in CIHP-1 cell lines and unilateral kidney injury model

C

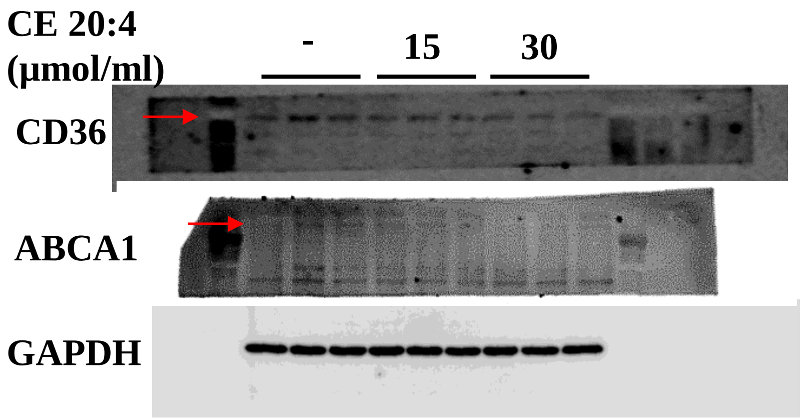

D

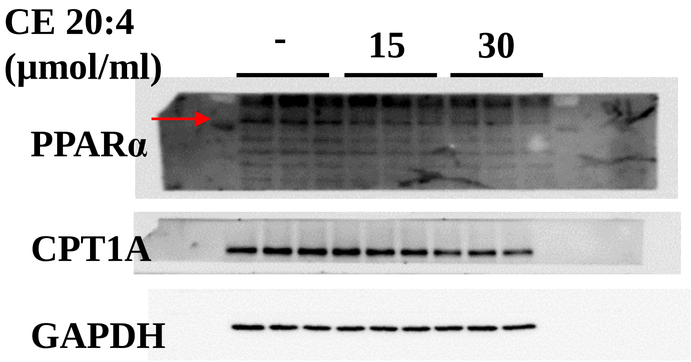

E

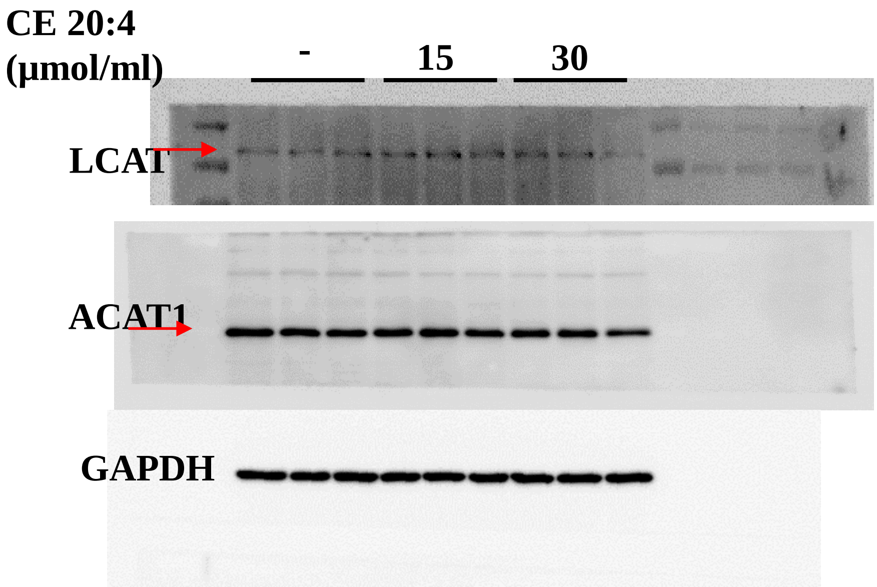

Figure 3. Lipid Accumulation and Changes in lipid metabolism factors in CIHP-1 cell lines and unilateral kidney injury model

F

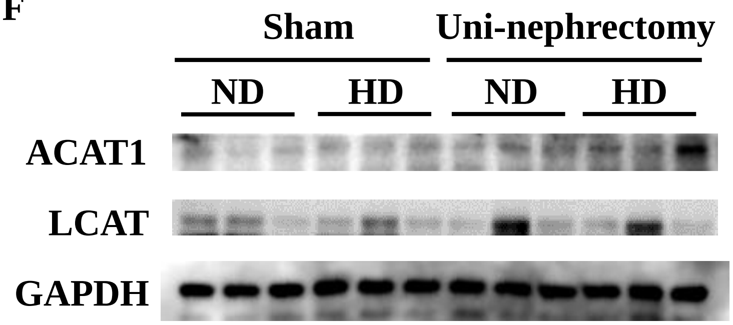

G

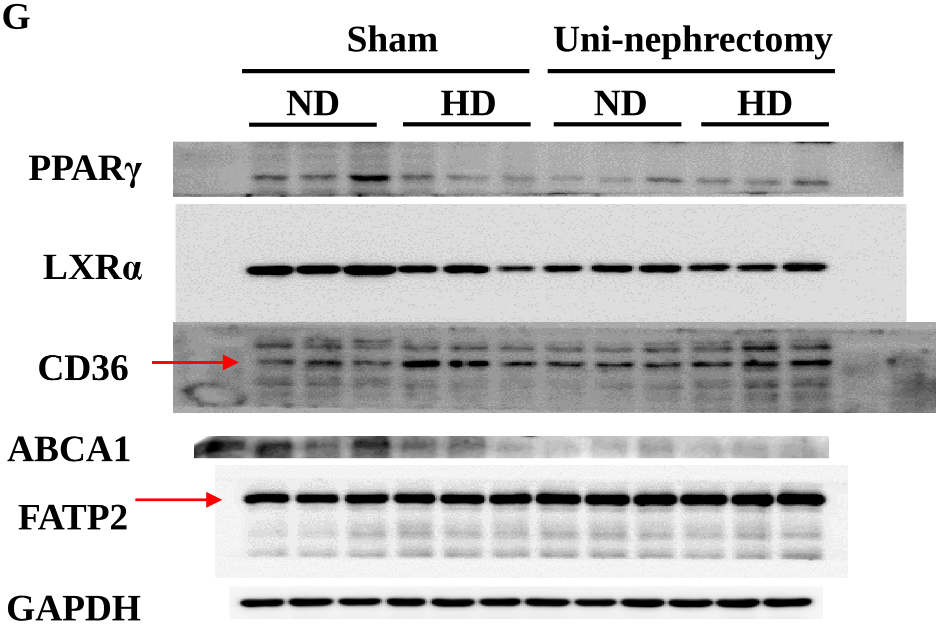

H

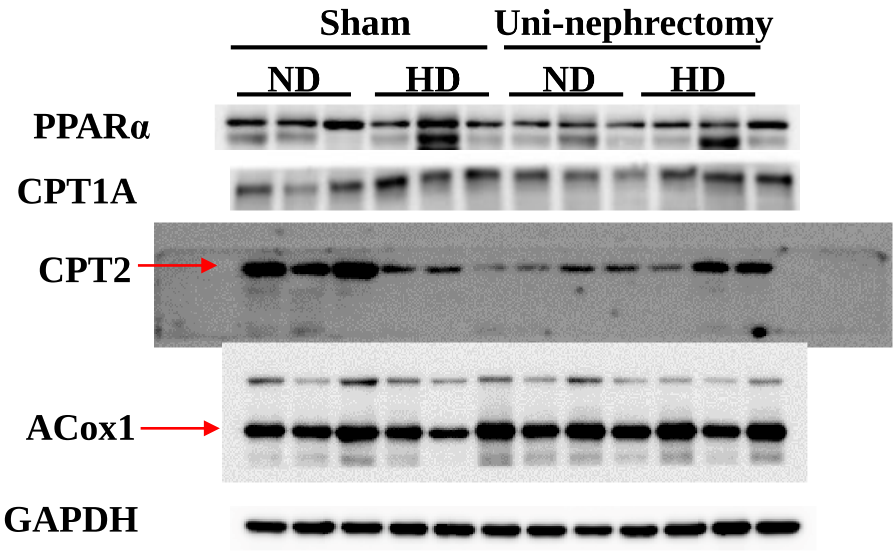

Figure 4. Mitochondrial damage caused by altered lipid metabolism in the CIHP-1 cell line.

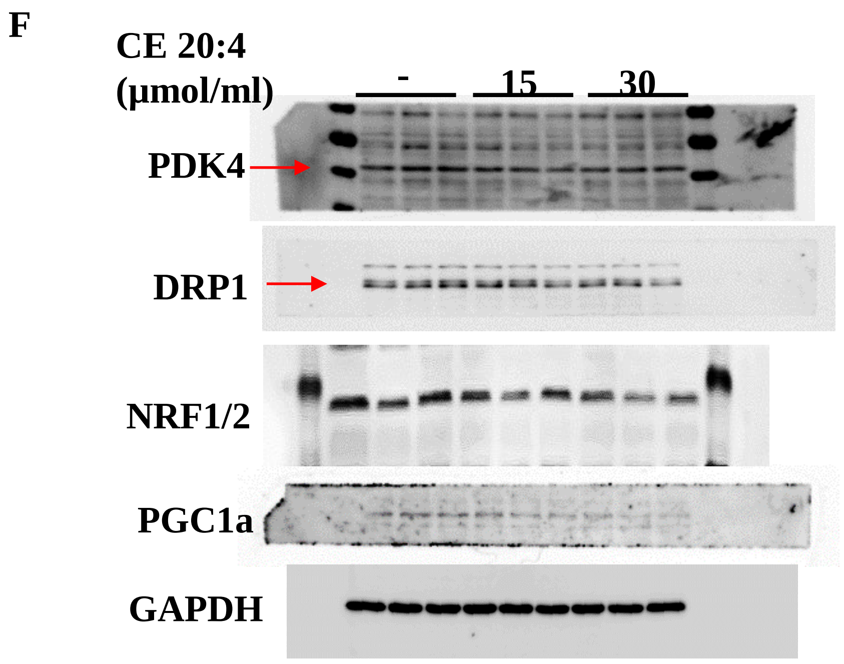

Figure 5. Mitochondrial damage caused by altered lipid metabolism in the unilateral kidney injury model.

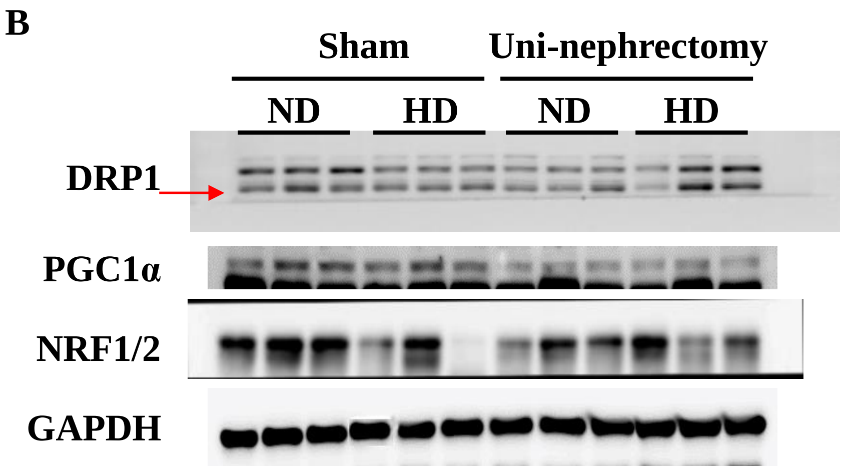

Figure 6. Autophagy activation by lipid accumulation and mitochondrial damage in the CIHP-1 cell line and unilateral renal injury model.

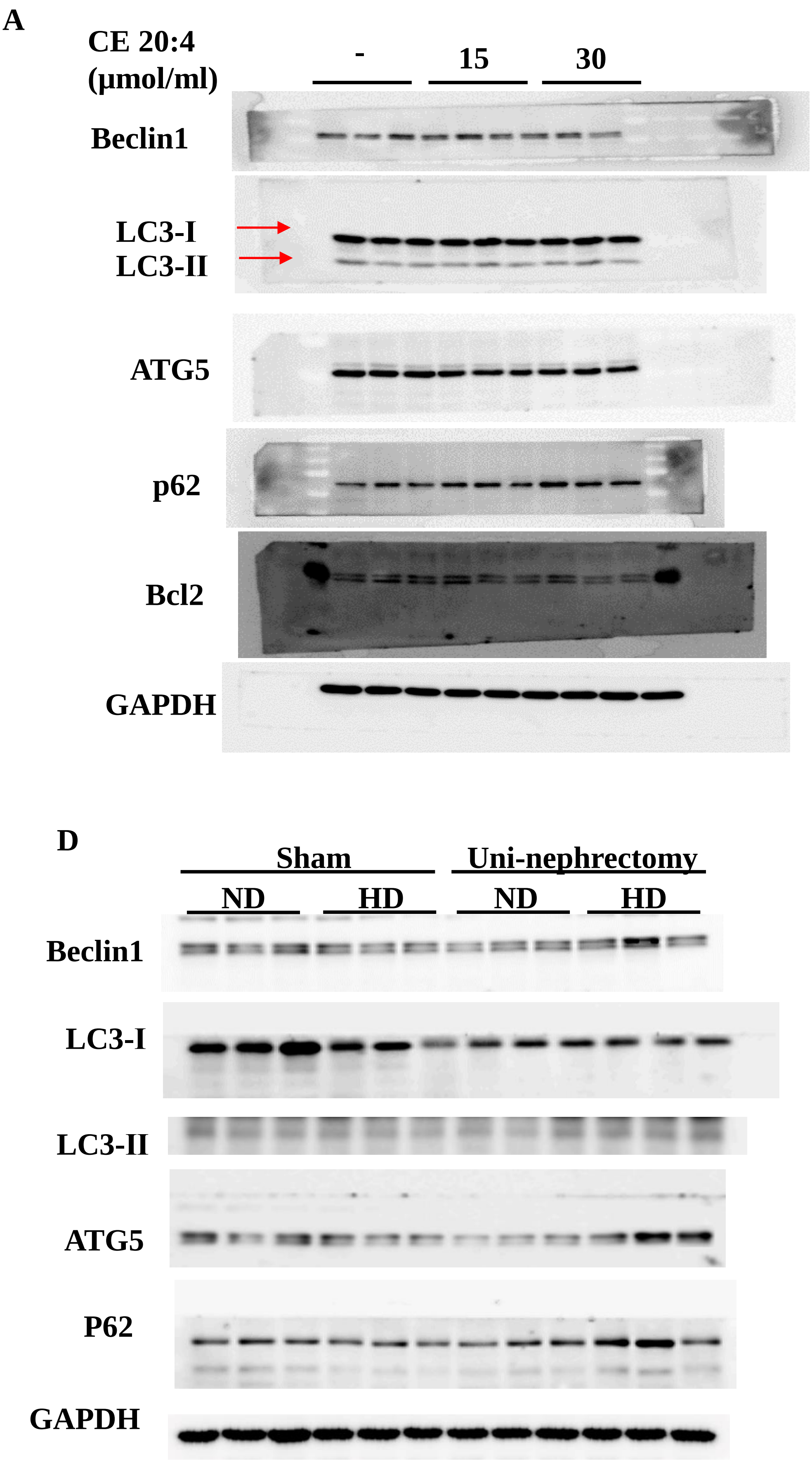

Figure 8. Confirmation of pathological damage, renal fibrosis due to changes in lipid metabolism in the unilateral renal injury model.

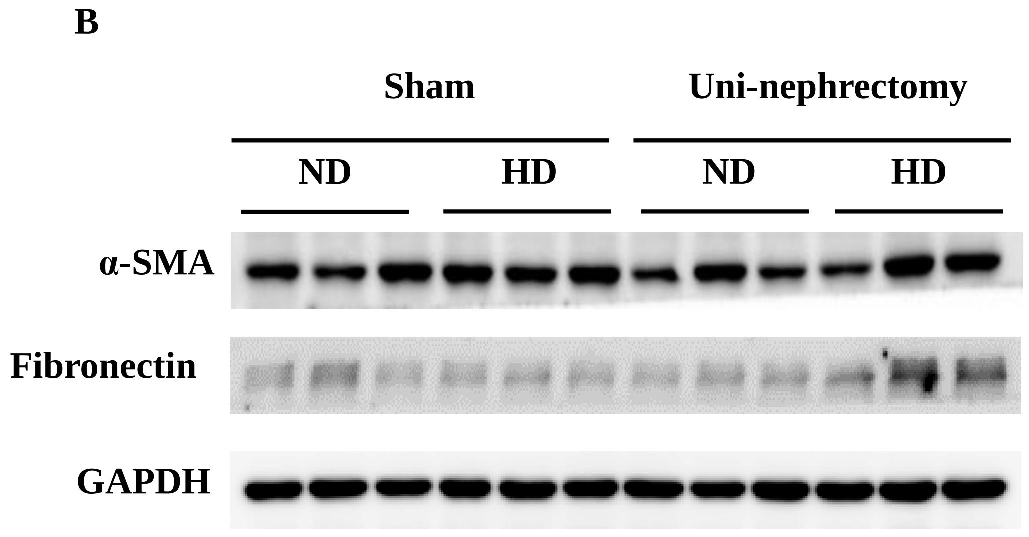

Supplement: Supplementary file 2 — Western blot raw band [file 41420_2025_2419_MOESM2_ESM.pdf]
